# Supplementary material for: Antidiabetic and cardiovascular beneficial effects of a liver-localized mitochondrial uncoupler
Source: Nat Commun. 2019 May 15;10:2172. doi: 10.1038/s41467-019-09911-6 (PMC6520346; doi:10.1038/s41467-019-09911-6)
Supplement: Supplementary file 1 — Supplementary Information [file 41467_2019_9911_MOESM1_ESM.pdf]

Supplementary information for

Antidiabetic and cardiovascular beneficial effects of a liver-localized mitochondrial uncoupler

Kanemoto et al.

Table of Contents

Supplementary Figures

Supplementary Figure 1: In vitro activity of OPC-163493 related to mUncoupling and a mitochondrial swelling assay using FCCP.
Supplementary Figure 2: PK and QWBA studies.
Supplementary Figure 3: Effects of OPC-163493 in multiple animal models.
Supplementary Figure 4: Blood glucose levels during hyperinsulinemic-euglycemic clamp test.
Supplementary Figure 5: Metabolomic analysis of in vivo effects of OPC-163493 in ZDF rats.
Supplementary Figure 6: Beneficial effects of OPC-163493 in salt-loaded SHRSPs.
Supplementary Figure 7: Isometric tension recordings obtained from rat thoracic aortas.
Supplementary Figure 8: Efficacy and toxicity of OPC-163493 in rats.
Supplementary Figure 9: Schemes of compound injections and OCR, CDER and ECAR measuring points.

Supplementary Tables

Supplementary Table 1: Radioactivity in tissues after single oral administration of [14C]-OPC-163493 suspension at 1 mg/kg to male rats by QWBA method
Supplementary Table 2: Radioactivity in tissues after single oral administration of [14C]-OPC-163493 suspension at 1 mg/kg to Day 17 pregnancy rats by QWBA method
Supplementary Table 3: Pharmacokinetic parameters of radioactivity in the plasma and liver following single oral administration of 1 mg/kg 14C-OPC-163493 in fasted male rats
Supplementary Table 4: HbA1c baseline of ZDF rats at the age of 11 weeks
Supplementary Table 5: Pharmacokinetic parameters of OPC-163493 in ZDF rats
Supplementary Table 6: HbA1c baseline of Akita mice at the age of 6 weeks
Supplementary Table 7: The Diurnal change of plasma OPC-163493 concentrations in Akita mice following 6-week dietary administration
Supplementary Table 8: HbA1c baseline of ZDF rats at the age of 27 weeks
Supplementary Table 9: Pharmacokinetic parameters of OPC-163493 in old ZDF rats
Supplementary Table 10: Plasma OPC-163493 concentrations at 6 AM in OLETF rats following 1-week dietary administration
Supplementary Table 11: The diurnal change of plasma OPC-163493 concentrations in ZDF(M) rats following 4-week dietary administration
Supplementary Table 12: HbA1c baseline of ob/ob mice at the age of 8 weeks

Supplementary Table 13: Plasma OPC-163493 concentrations at 6 AM in ob/ob mice following 10-week dietary administration
Supplementary Table 14: Lipidomic analysis of effects of OPC-163493 on hepatic lipids in HFD SD rats
Supplementary Table 15: Pharmacokinetic parameters of OPC-163493 in HDF SD rats
Supplementary Table 16: Long-term effect of OPC-163493 dosing with mixed chow on HbA1c values in male OLETF rats
Supplementary Table 17: Effects of OPC-163493 on hyperinsulinemic-euglycemic clamp parameters
Supplementary Table 18: Metabolomic analysis of in vitro effects of OPC-163493 in Hep G2 cells
Supplementary Table 19: Pharmacokinetic parameters of OPC-163493 in ZDF rats
Supplementary Table 20: Metabolomic analysis of in vivo effects of OPC-163493 in the liver of ZDF rats
Supplementary Table 21: Parameters of OPC-163493 intervention study in salt-loaded SHRSPs (1st exp.)
Supplementary Table 22: Parameters of OPC-163493 intervention study in salt-loaded SHRSPs (2nd exp.)
Supplementary Table 23: Sensitization of OPC-163493 to NP-induced relaxation of rat aortas
Supplementary Table 24: Summary of 4-week repeated oral dosing toxicity study in rats
Supplementary Table 25: Summary of 13-week repeated oral dosing toxicity study in rats
Supplementary Table 26: Toxic changes observed with a four-week, repeated oral dosing toxicity study in rats
Supplementary Table 27: Inhibitory effect of OPC-163493 on radioligand binding to various receptors, ion channels and transporters

Supplementary Discussions

Supplementary Discussion 1
Supplementary Discussion 2

Supplementary References

Supplementary Figures

a

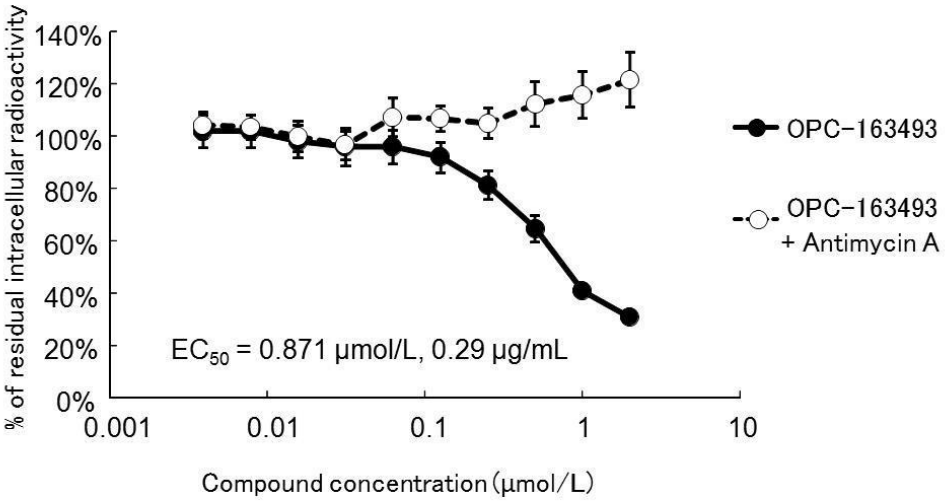

b

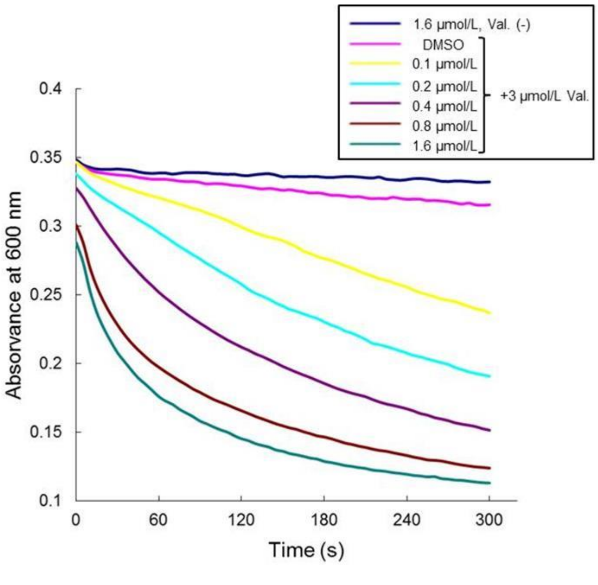

c

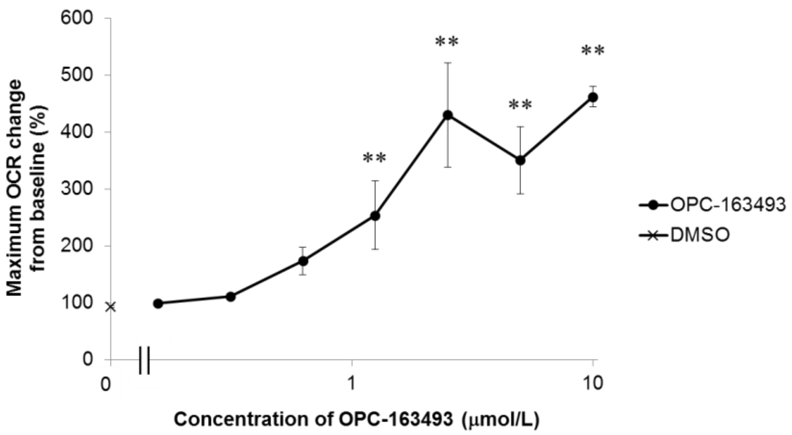

d

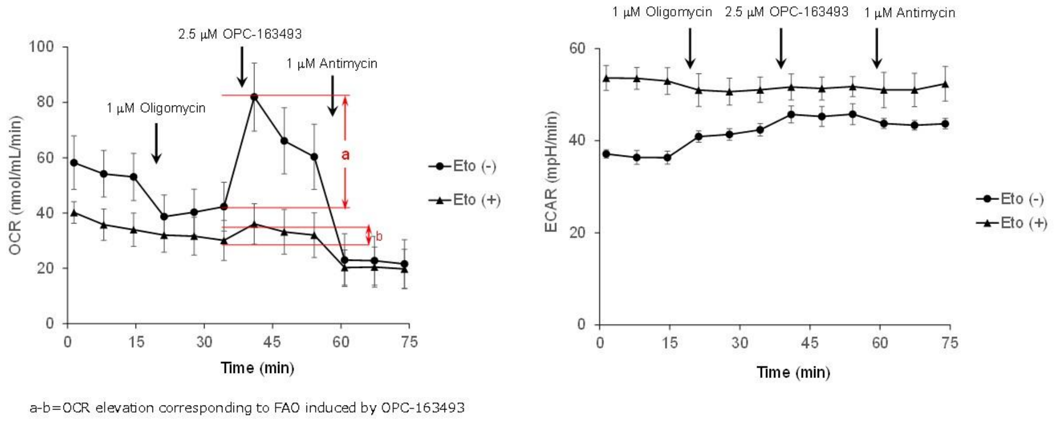

Supplementary Figure 1 In vitro activity of OPC-163493 related to mUncoupling and a mitochondrial swelling assay using FCCP. (a) Assay of TCA cycle activation by OPC-163493 in NaCT stably expressed CHO (NaCT-CHO) cells. Data represent mean ± SE (n = 4). EC_{50} = 0.871 (95% CI: 0.667–1.138) μM, estimated by Logit regression analysis (compound concentrations were subjected to logarithmic transformation). The activity disappeared on addition of 0.1 μM antimycin A. (**b**) Mitochondrial swelling assay of isolated mitochondria from the liver of a male SD rat. The indicated concentration of FCCP was added to isolated mitochondrial solution in isotonic acetate buffer with or without valinomycin (Val) at 0 sec and absorbance of the solution was measured at 600 nm. Dark blue line; 1.6 μM without Val, pink line; DMSO control, yellow line; 0.1 μM with Val, blue line; 0.2 μM with Val, purple line; 0.4 μM with Val, brown line; 0.8 μM with Val, green line; 1.6 μM with Val. (**c**) OCR measurement in rat primary hepatocytes. Data represent mean ± SE (n = 5). **: p<0.01, OPC vs DMSO control using a two-tailed Williams' test. (**d**) Effect of OPC-163493 on OCR and ECAR in HepG2 cells with glucose-depleted medium. Data represent mean ± SE (n = 3). Eto: etomoxir.

a

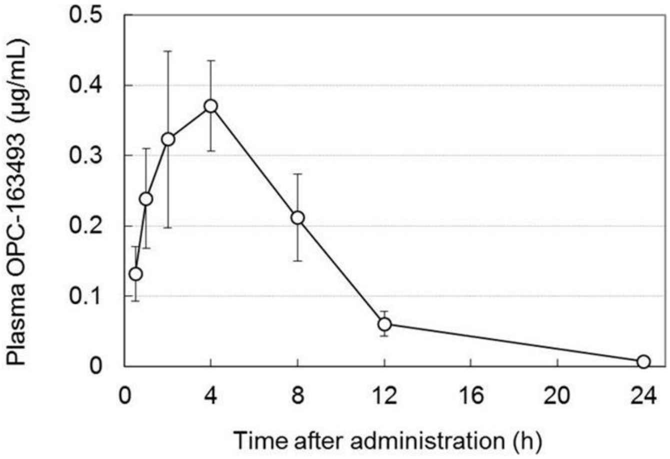

Pharmacokinetics of oral OPC-163493

C_{max} (μg/mL)	0.3929
t_{max} (h)	3.5
$t_{1/2}$ (h)	3.735
BA (%)	53.50

b

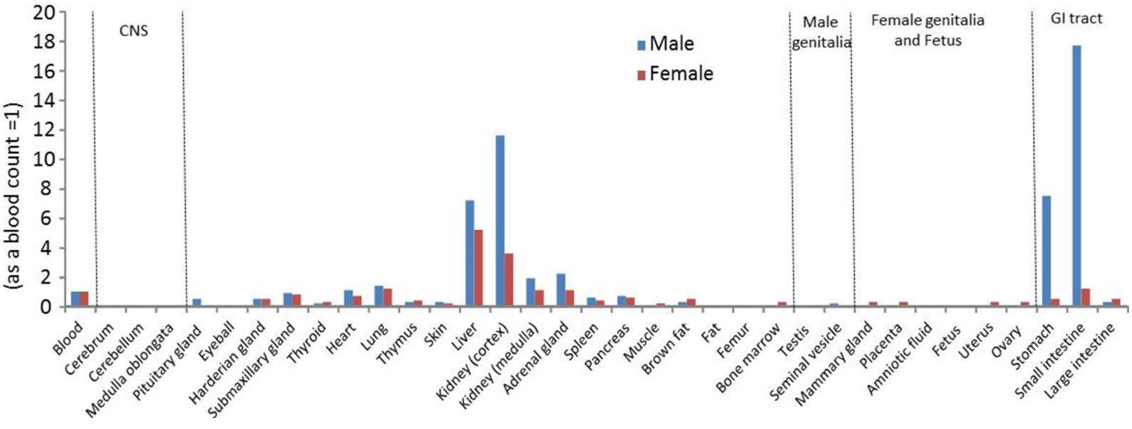

c

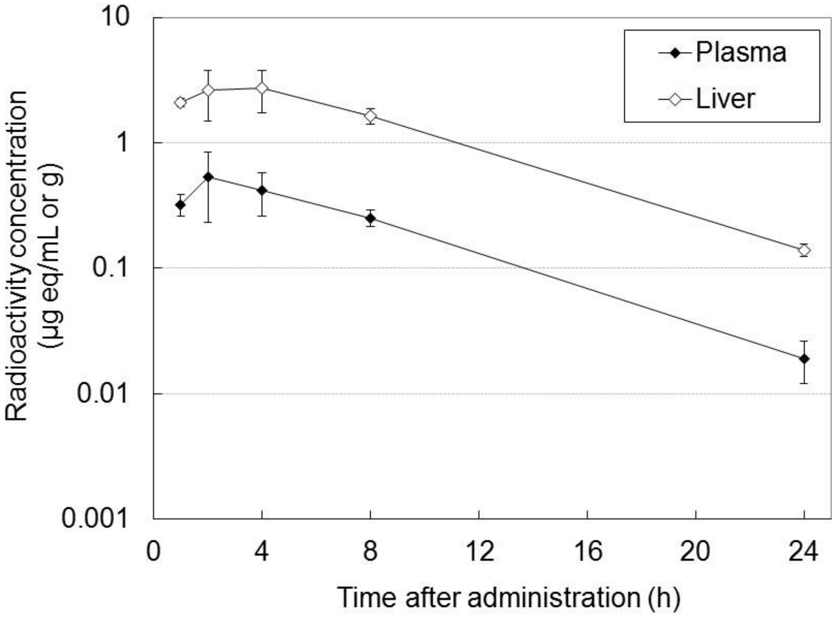

Supplementary Figure 2 PK and QWBA studies. (a) OPC-163493 plasma concentrations following single oral administration (1 mg kg^{-1}) to fasted male SD rats, with PK parameters. Data represent mean ± SD (n = 4). (b) Relative radioactivity in tissues 2 h after single oral administration of [^{14}C]-OPC-163493 suspension at 1 mg kg^{-1} to rats using the QWBA method. Relative ratios are shown using blood radioactivity as a reference value of 1.0. (c) Plasma and liver concentration of radioactivity following single oral administration of 1 mg kg^{-1} [^{14}C]-OPC-163493 in fasted male rats. Data represent mean ± SD (n = 3).

a

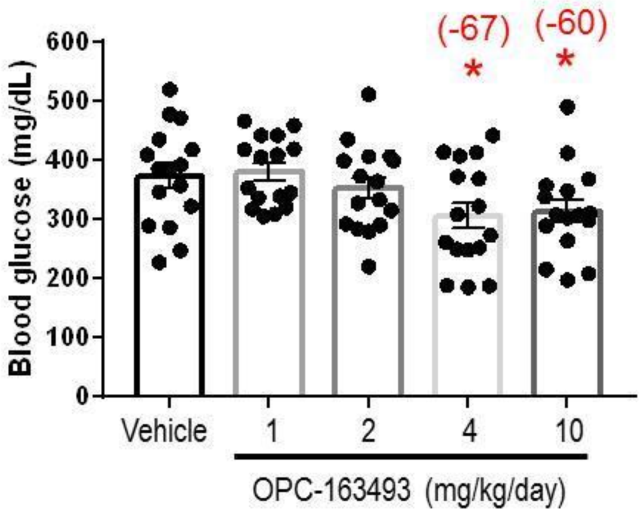

(-67)
(-60)
*
*
600
500
400
300
200
100
0
Blood glucose (mg/dL)
Vehicle
1
2
4
10
OPC-163493 (mg/kg/day)

b

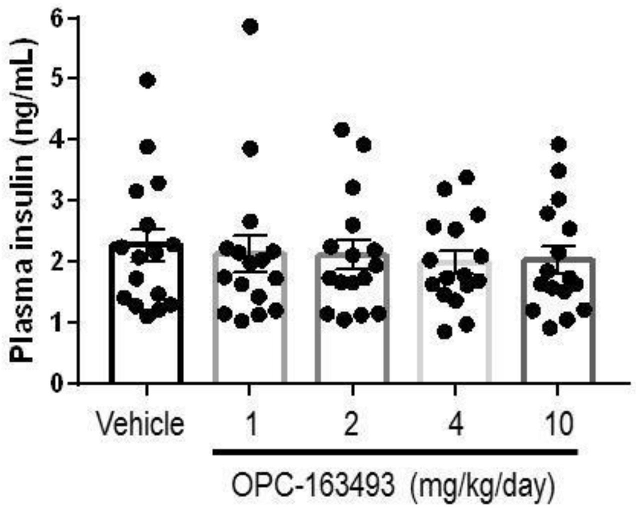

6
5
4
3
2
1
0
Plasma insulin (ng/mL)
Vehicle
1
2
4
10
OPC-163493 (mg/kg/day)

c

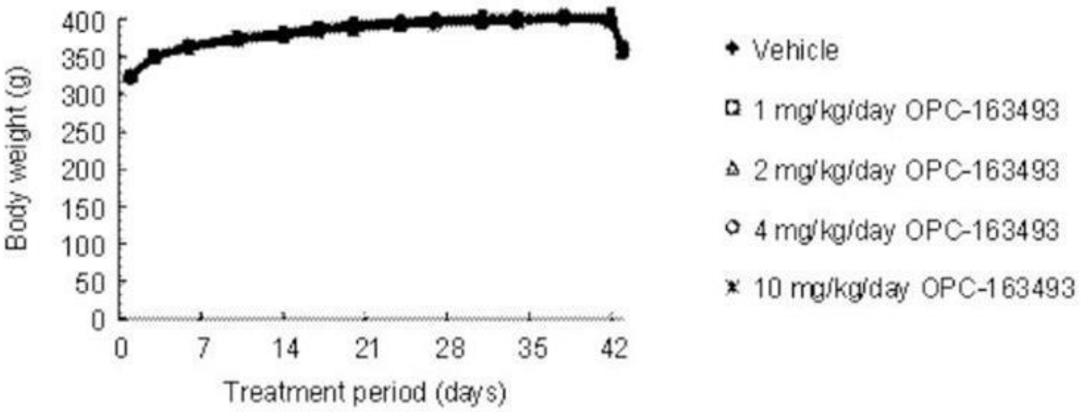

400
350
300
250
200
150
100
50
0
Body weight (g)
0
7
14
21
28
35
42
Treatment period (days)
Vehicle
1 mg/kg/day OPC-163493
2 mg/kg/day OPC-163493
4 mg/kg/day OPC-163493
10 mg/kg/day OPC-163493

d

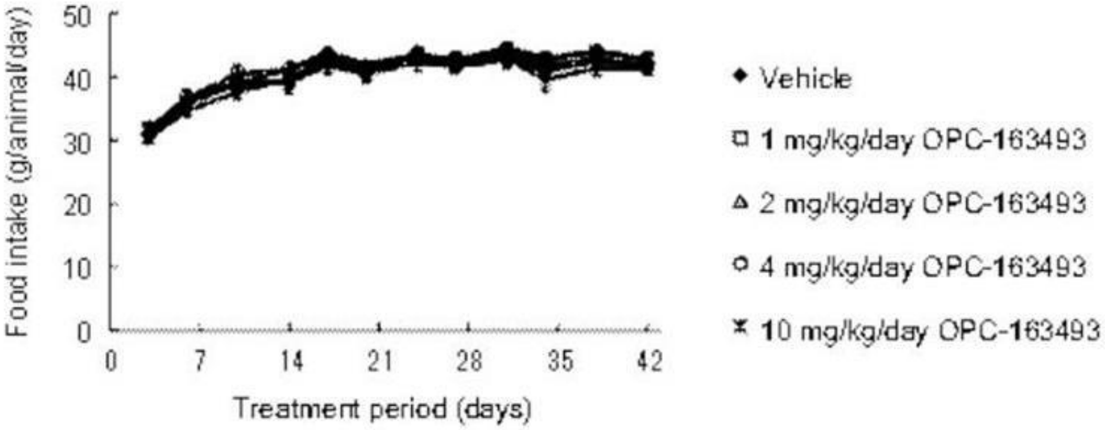

Food intake (g/animal/day)
50
40
30
20
10
0
0
7
14
21
28
35
42
Treatment period (days)
Vehicle
1 mg/kg/day OPC-163493
2 mg/kg/day OPC-163493
4 mg/kg/day OPC-163493
10 mg/kg/day OPC-163493

e

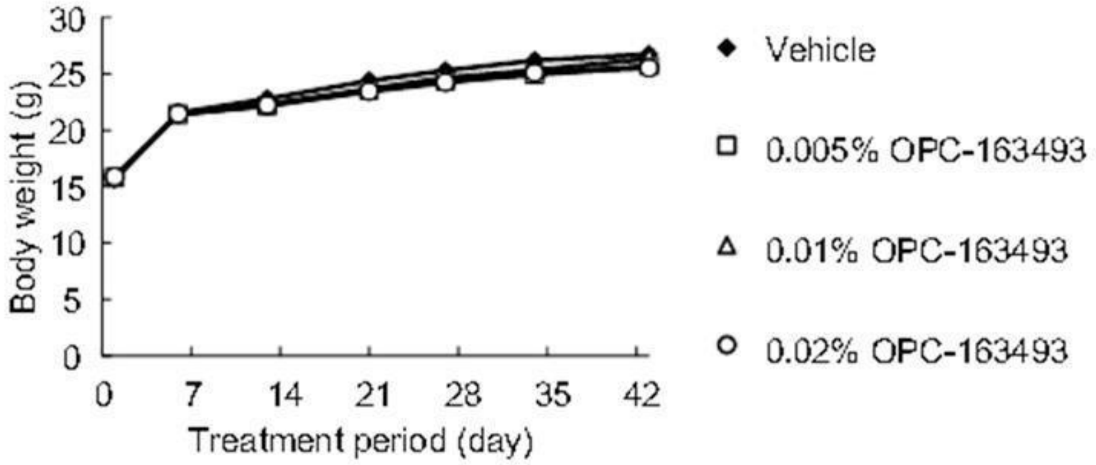

Body weight (g)
30
25
20
15
10
5
0
0
7
14
21
28
35
42
Treatment period (day)
Vehicle
0.005% OPC-163493
0.01% OPC-163493
0.02% OPC-163493

f

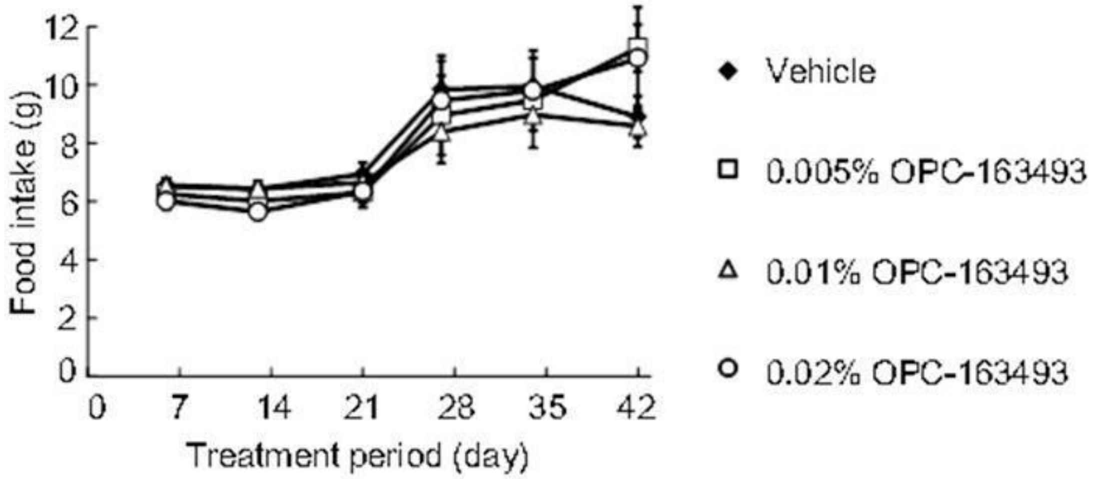

Food intake (g)
12
10
8
6
4
2
0
0
7
14
21
28
35
42
Treatment period (day)
Vehicle
0.005% OPC-163493
0.01% OPC-163493
0.02% OPC-163493

g

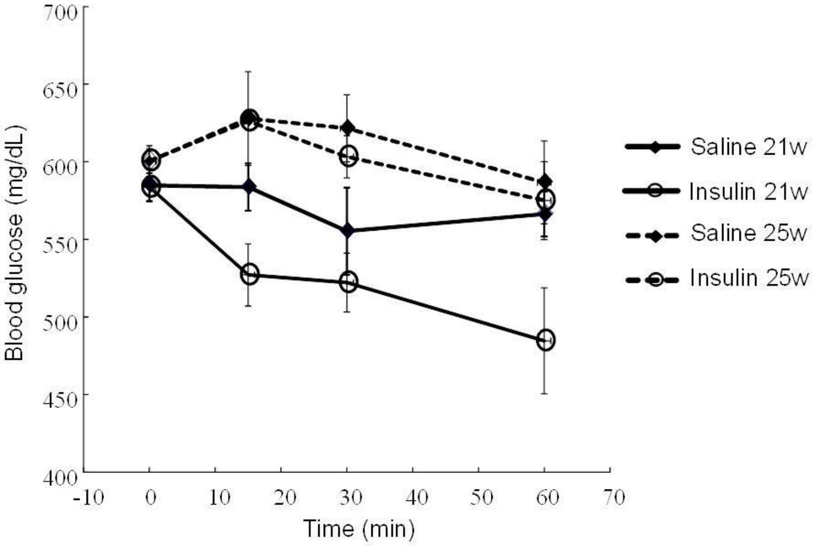

700
650
600
550
500
450
400
Blood glucose (mg/dL)
-10
0
10
20
30
40
50
60
70
Time (min)
Saline 21w
Insulin 21w
Saline 25w
Insulin 25w

h

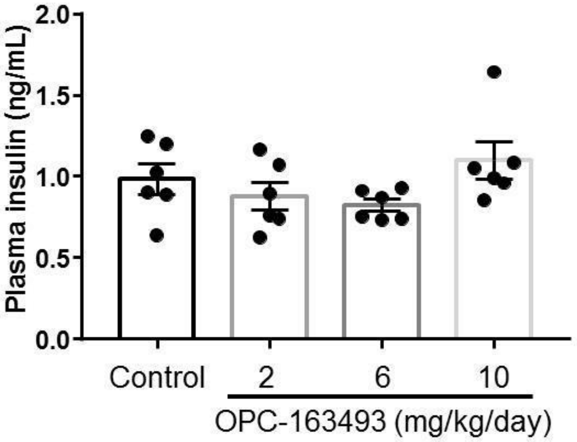

2.0
1.5
1.0
0.5
0.0
Plasma insulin (ng/mL)
Control
2
6
10
OPC-163493 (mg/kg/day)

i

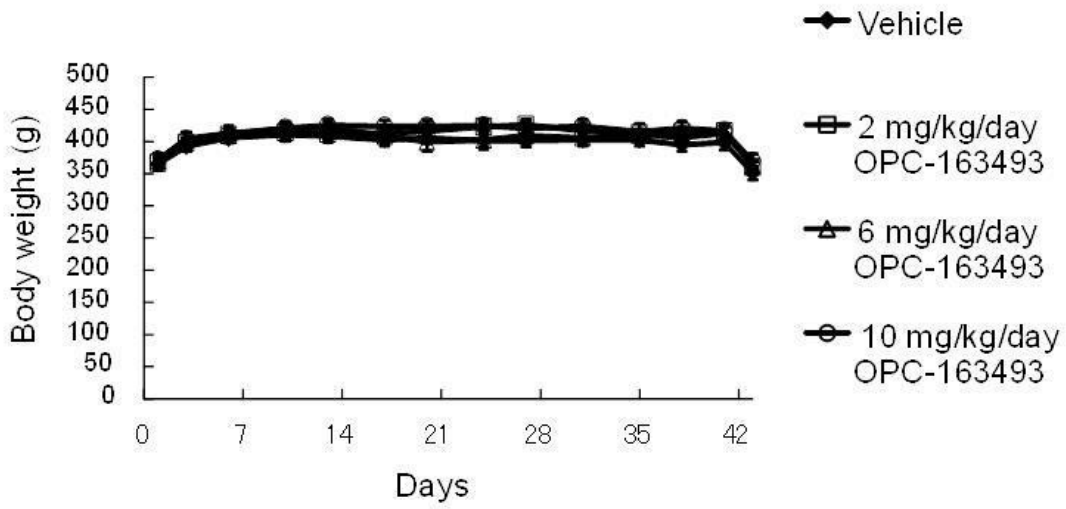

Vehicle
2 mg/kg/day OPC-163493
6 mg/kg/day OPC-163493
10 mg/kg/day OPC-163493
Body weight (g)
500
450
400
350
300
250
200
150
100
50
0
0
7
14
21
28
35
42
Days

j

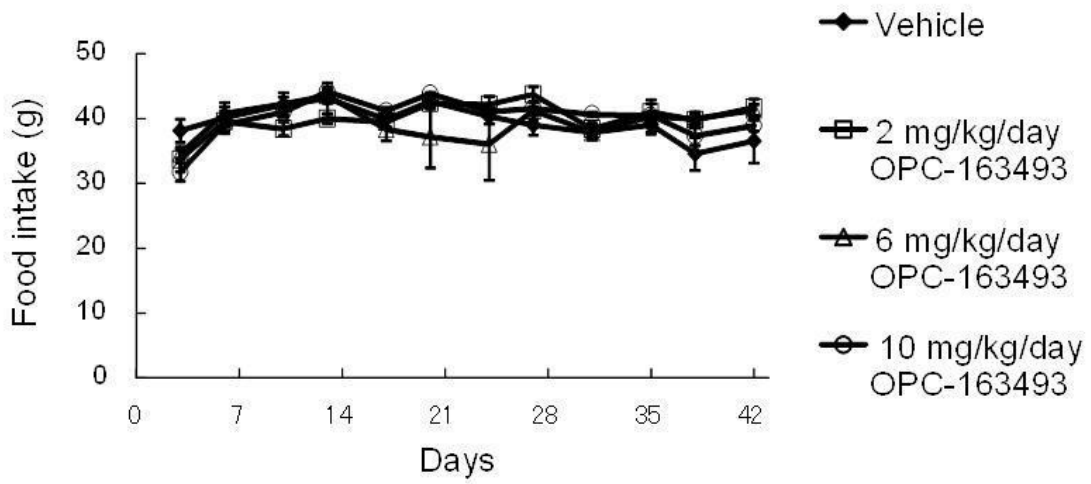

Vehicle
2 mg/kg/day OPC-163493
6 mg/kg/day OPC-163493
10 mg/kg/day OPC-163493
Food intake (g)
50
40
30
20
10
0
0
7
14
21
28
35
42
Days

k

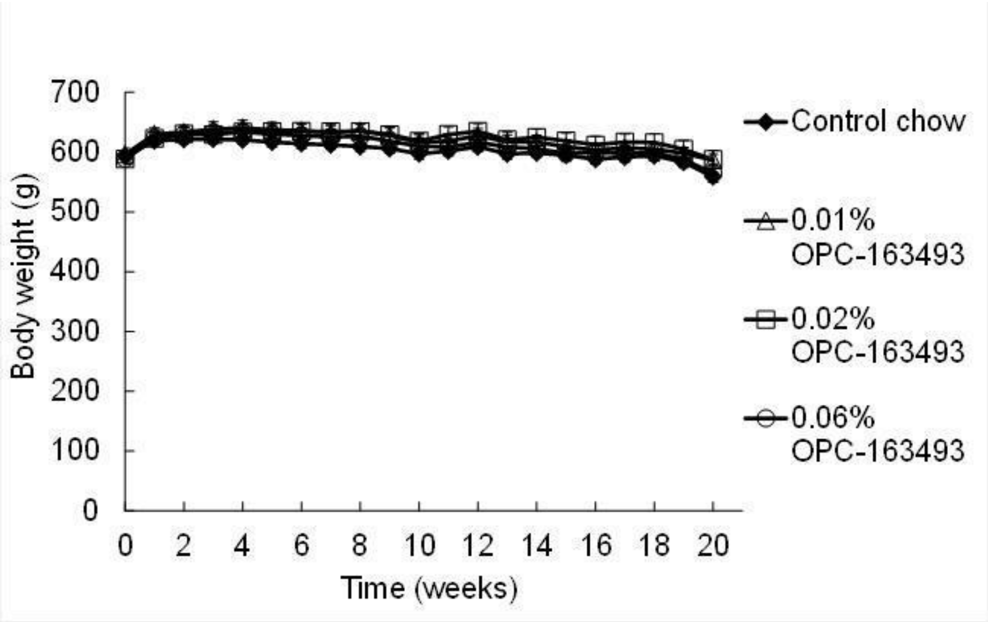
700
600
500
400
300
200
100
0
Body weight (g)
0 2 4 6 8 10 12 14 16 18 20
Time (weeks)
Control chow
0.01% OPC-163493
0.02% OPC-163493
0.06% OPC-163493

l

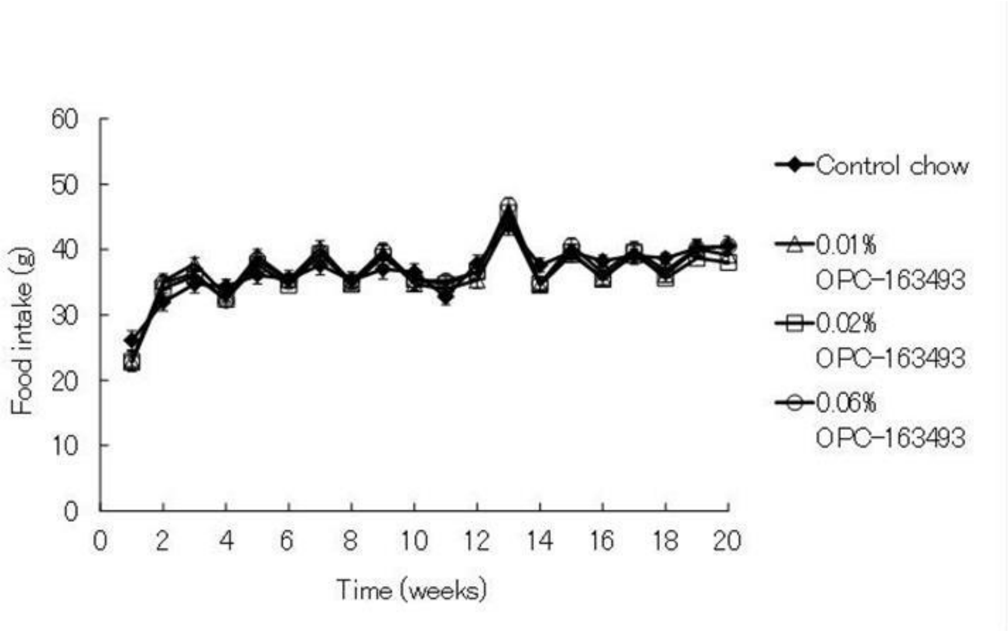
60
50
40
30
20
10
0
Food intake (g)
0 2 4 6 8 10 12 14 16 18 20
Time (weeks)
Control chow
0.01% OPC-163493
0.02% OPC-163493
0.06% OPC-163493

m

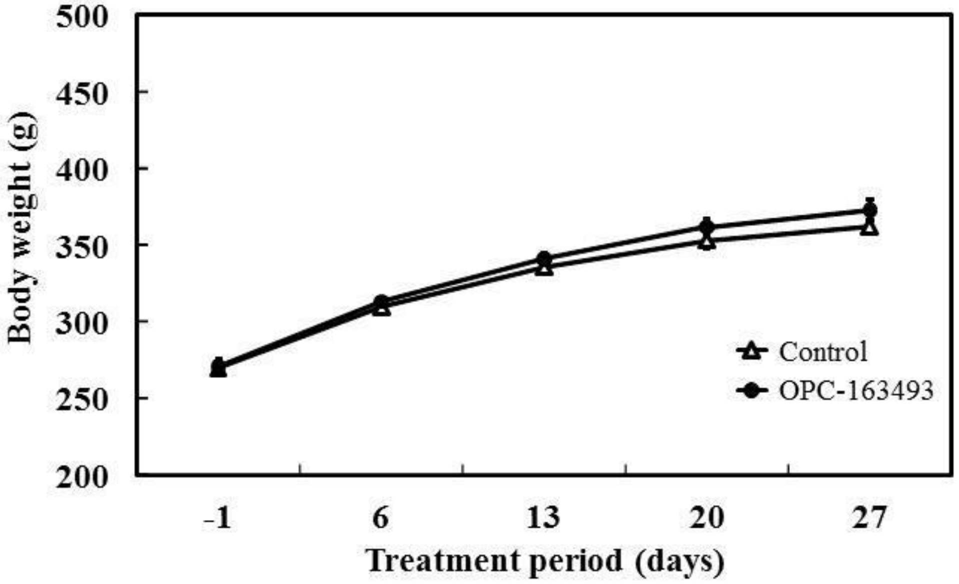
500
450
400
350
300
250
200
Body weight (g)
-1
6
13
20
27
Treatment period (days)
Control
OPC-163493

n

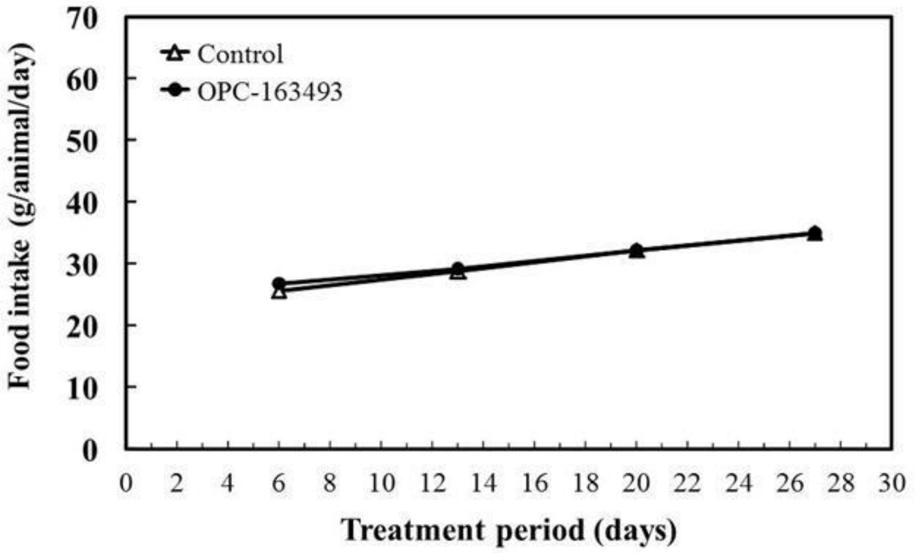
70
60
50
40
30
20
10
0
Food intake (g/animal/day)
0 2 4 6 8 10 12 14 16 18 20 22 24 26 28 30
Treatment period (days)
Control
OPC-163493

o

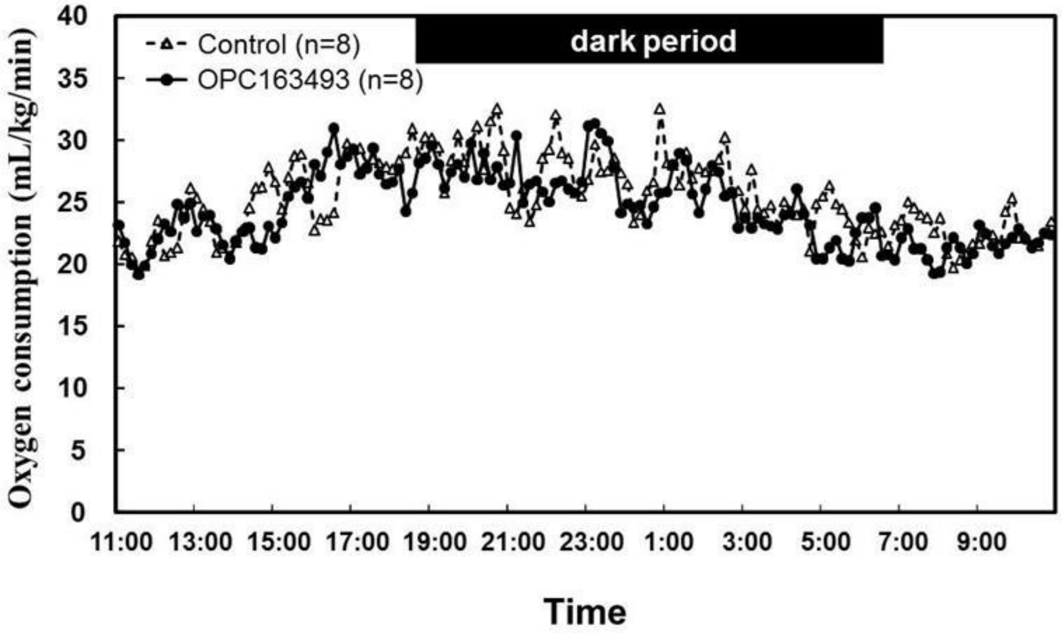

Control (n=8)
OPC163493 (n=8)
dark period
Oxygen consumption (mL/kg/min)
0
5
10
15
20
25
30
35
40
11:00
13:00
15:00
17:00
19:00
21:00
23:00
1:00
3:00
5:00
7:00
9:00
Time

p

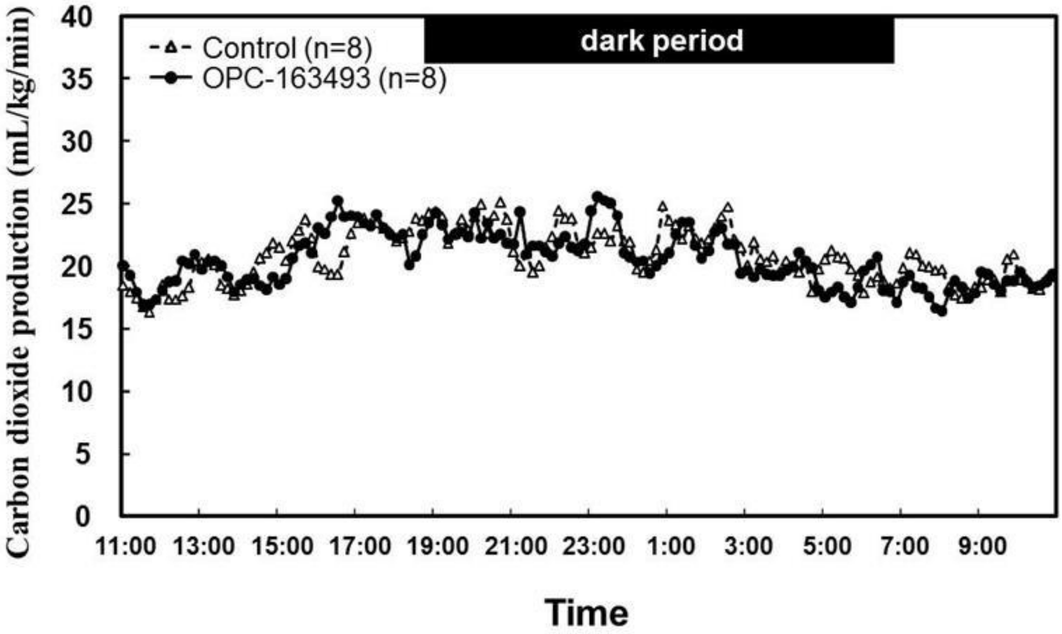

Control (n=8)
OPC-163493 (n=8)
dark period
Carbon dioxide production (mL/kg/min)
0
5
10
15
20
25
30
35
40
11:00
13:00
15:00
17:00
19:00
21:00
23:00
1:00
3:00
5:00
7:00
9:00
Time

q

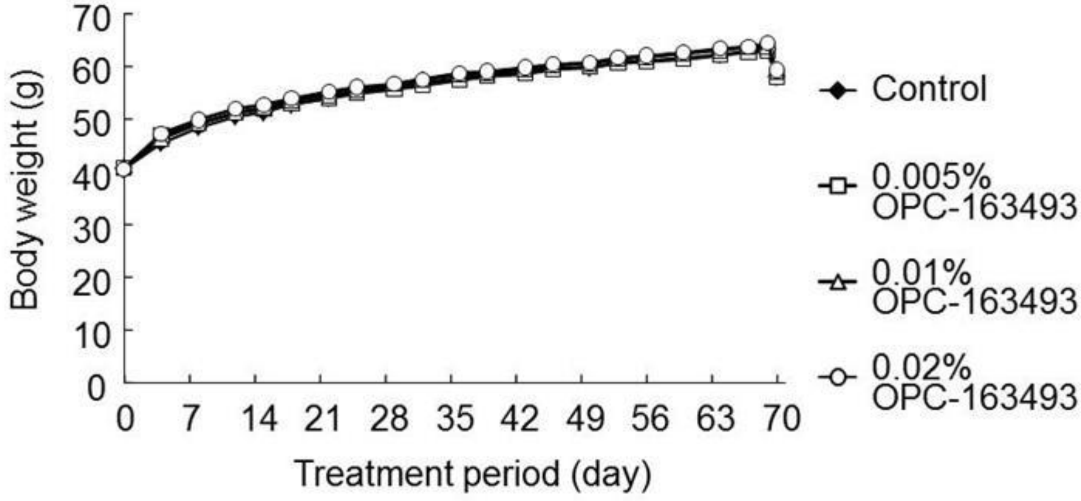
Body weight (g)
70
60
50
40
30
20
10
0
0 7 14 21 28 35 42 49 56 63 70
Treatment period (day)
Control
0.005% OPC-163493
0.01% OPC-163493
0.02% OPC-163493

r

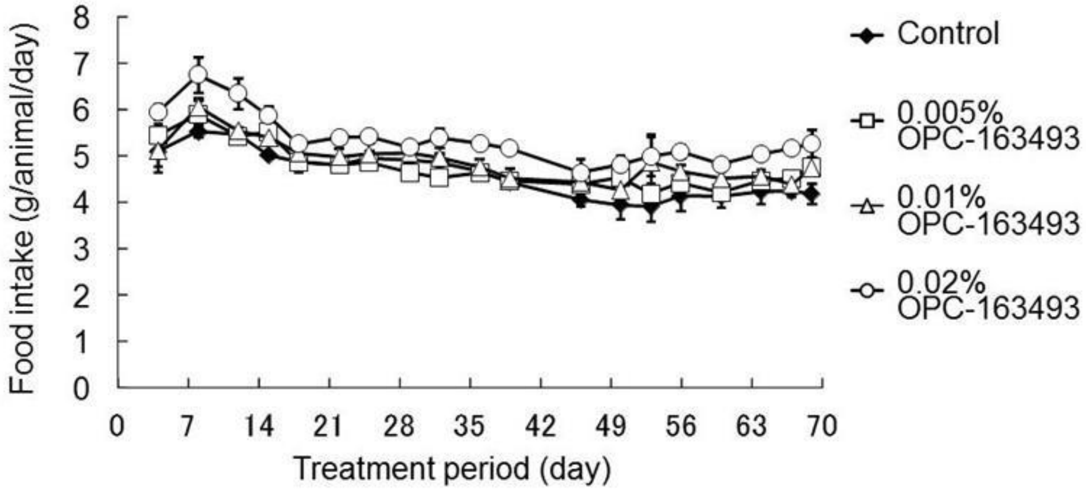
Food intake (g/animal/day)
8
7
6
5
4
3
2
1
0
0 7 14 21 28 35 42 49 56 63 70
Treatment period (day)
Control
0.005% OPC-163493
0.01% OPC-163493
0.02% OPC-163493

s

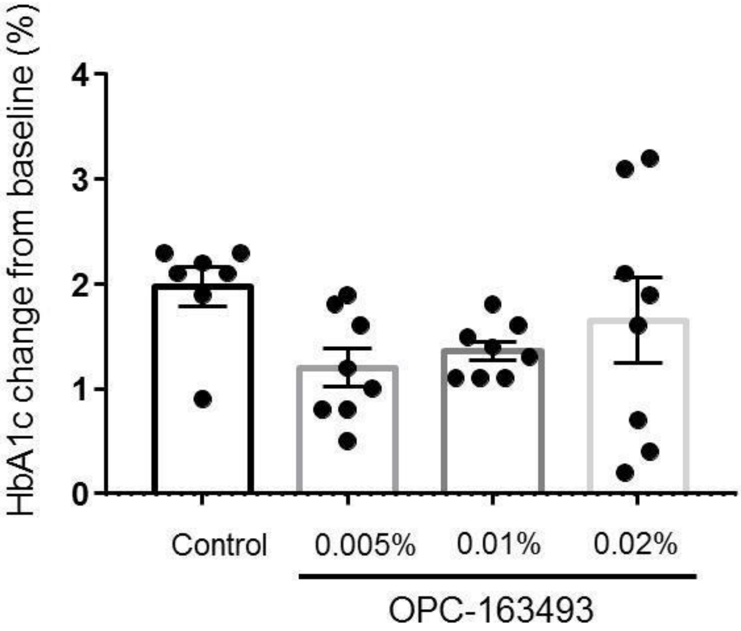

HbA1c change from baseline (%)
4
3
2
1
0
Control
0.005%
0.01%
0.02%
OPC-163493

t

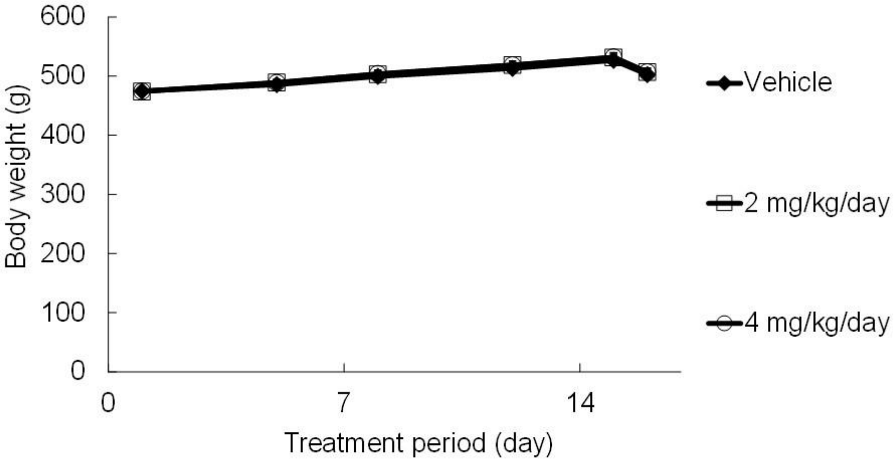

Body weight (g)
600
500
400
300
200
100
0
0
7
14
Treatment period (day)
Vehicle
2 mg/kg/day
4 mg/kg/day

u

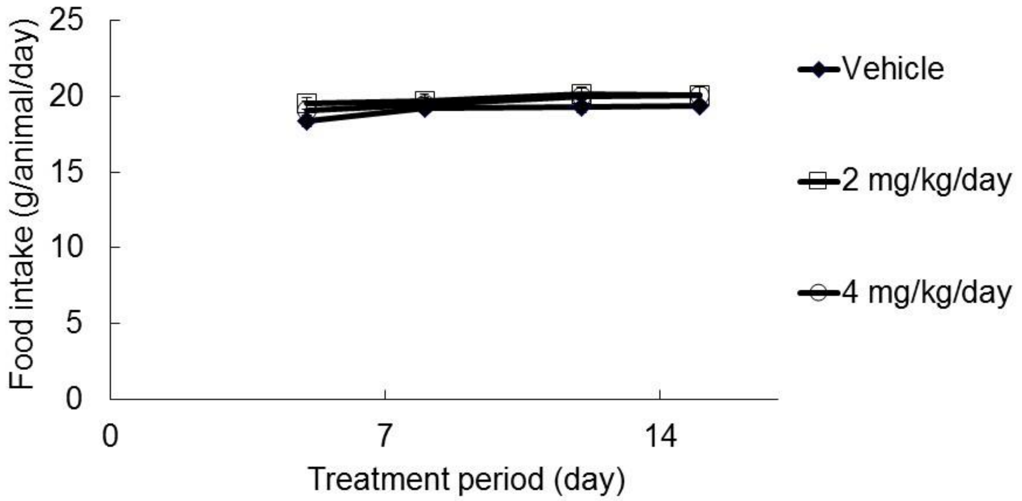

Food intake (g/animal/day)
25
20
15
10
5
0
0
7
14
Treatment period (day)
Vehicle
2 mg/kg/day
4 mg/kg/day

v

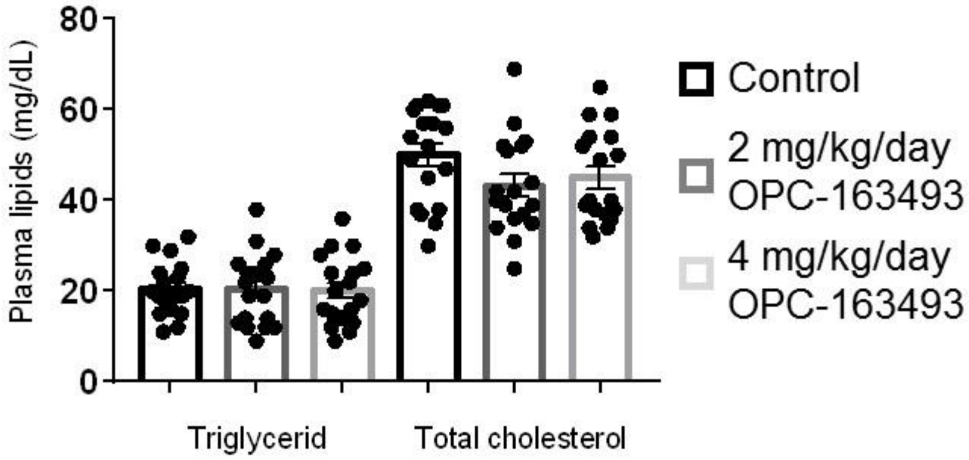

Plasma lipids (mg/dL)
80
60
40
20
0
Triglycerid
Total cholesterol
Control
2 mg/kg/day OPC-163493
4 mg/kg/day OPC-163493

w

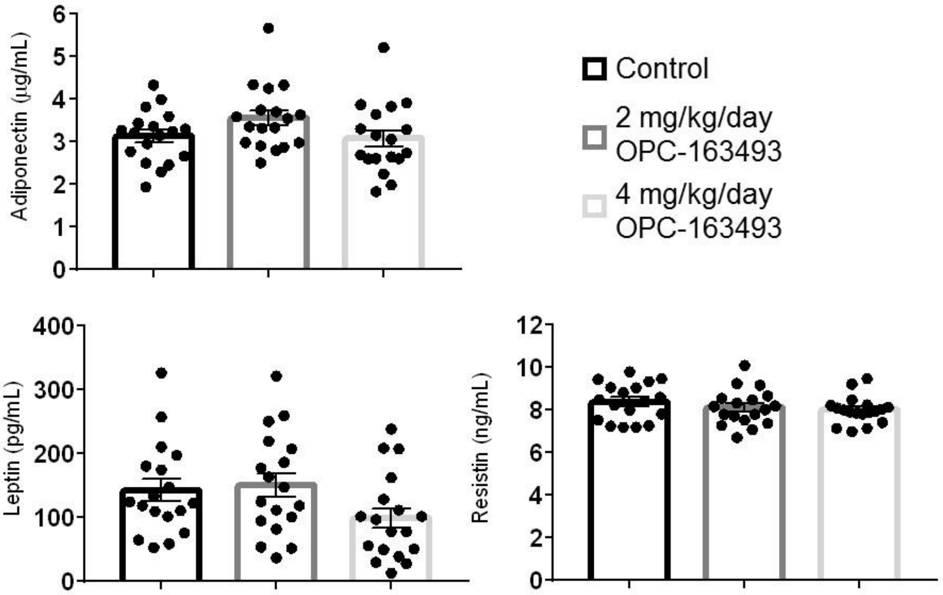

Supplementary Figure 3 Effects of OPC-163493 in multiple animal models. (a) Effect of OPC-163493 on fasting blood glucose levels in ZDF rats. Data represent mean ± SE (n = 16). Significant efficacy was found in the 4 and 10 mg kg^{-1} day^{-1} OPC-163493-treated groups (*P < 0.05, OPC vs Vehicle group by Williams' test with two-way ANOVA). (b) Effect of OPC-163493 on fasting insulin level in ZDF rats. Data represent mean ± SE (n = 16). No significant difference was found using Williams' test with two-way ANOVA. (c) Effect of OPC-163493 on body weight change in ZDF rats. Data represent mean ± SE (n = 16). No significant differences were found using the MMRM method followed by Dunnett's test. Fasting was performed between Days 42 and 43. (d) Effect of OPC-163493 on the change in food intake of ZDF rats. Data represent mean ± SE (n = 8). Food intake data are presented as the average values per animal per day. Two animals were accommodated in each cage. No significant differences were found using the MMRM method followed by Dunnett's test. (e) Effect of OPC-163493 on body weight change in Akita mice. Data represent mean ± SE (n = 12). No significant differences were found using the MMRM method. (f) Effect of OPC-163493 on food intake change in Akita mice. Data represent mean ± SE (n = 6). Two mice were accommodated in each cage. No significant differences were found using the MMRM method. (g) Insulin sensitivity of ZDF rats at the age of 21 and 25 weeks. Insulin tolerance tests were performed using old ZDF rats. One unit kg^{-1} insulin or saline were injected intraperitoneally at 0 min. Insulin marginally decreased the blood glucose of 21-week-old ZDF rats (not significant); the response finally disappeared in the rats at 25 weeks. Data represent mean ± SE (n = 6). No significant differences were found

in the time-dependent changes in blood glucose levels between saline and insulin-treated groups using the MMRM method. (h) Effect of OPC-163493 on fasting insulin level on Day 43 in old ZDF rats. Data represent mean ± SE (n = 6). No significant difference was found using a two-tailed Williams' test. (i) Effect of OPC-163493 on body weight change in old ZDF rats. Data represent mean ± SE (n = 6). No significant differences were found using the MMRM method. Fasting was performed between Days 42 and 43. (j) Effect of OPC-163493 on food intake change in old ZDF rats. Data represent mean ± SE (n = 3). Two rats were accommodated in each cage. No significant differences were found using the MMRM method. (k) Effect of OPC-163493 on body weight change in OLETF rats. Data represent mean ± SE (n = 14). No significant differences were found using the MMRM method. (l) Effect of OPC-163493 on food intake change in OLETF rats. Data represent mean ± SE (n = 14). No significant differences were found using the MMRM method. (m) Effect of OPC-163493 on body weight change in ZDF(M) rats. Data represent mean ± SE (n = 8). No significant differences were found using the MMRM method. (n) Effect of OPC-163493 on food intake change in ZDF(M) rats. Data represent mean ± SE (n = 8). No significant differences were found using the MMRM method. (o) Effect of OPC-163493 on oxygen consumption in ZDF(M) rats after four weeks of treatment. Data represent mean (n = 8). (p) Effect of OPC-163493 on carbon dioxide production in ZDF(M) rats after four weeks of treatment. Data represent mean (n = 8). (q) Effect of OPC-163493 on body weight change in ob/ob mice. Data represent mean ± SE (control group: n = 7, other groups: n = 8). No significant difference was found using the MMRM method. Fasting was enforced between Days 69 and 70. (r) Effect of OPC-163493 on food intake change in ob/ob mice. Data represent mean ± SE (n = 4). Two mice were accommodated in each cage. A significant difference was found between the control and 0.02% OPC-treatment groups using the MMRM method followed by Dunnett's test ($P < 0.01$). (s) Effect of OPC-163493 on HbA1c changes from baseline in ob/ob mice. Data represent mean ± SE (control group: n = 7, other groups: n = 8). No significant differences were found using Dunnett's test. (t) Effect of OPC-163493 on body weight change in HFD SD rats. Data represent mean ± SE (n = 18). No significant difference was found using the MMRM method followed by Dunnett's test. Fasting was performed between Day 15 and Day 16. (u) Effect of OPC-163493 on food intake change in HFD SD rats. Data represent mean ± SE (n = 9). No significant differences were found using the MMRM method followed by Dunnett's test. (v) Effect of OPC-163493 on plasma lipids in HFD SD rats. Data represent mean ± SE (n = 18). No significant differences were found by Dunnett's test. (w) Effect of OPC-163493 on plasma adipokines in HFD SD rats. Data represent mean ± SE (n = 18). No significant differences were found by Dunnett's test.

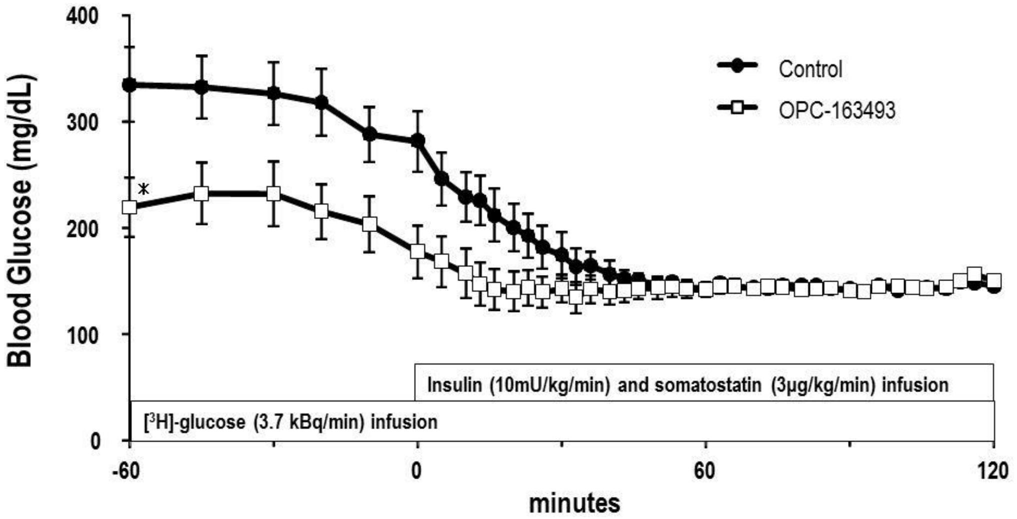

Supplementary Figure 4 Blood glucose levels during hyperinsulinemic-euglycemic clamp test. Data represent mean ± SE (n = 12). A significant difference was found at -60 min (* $P < 0.05$ by unpaired t-test).

a

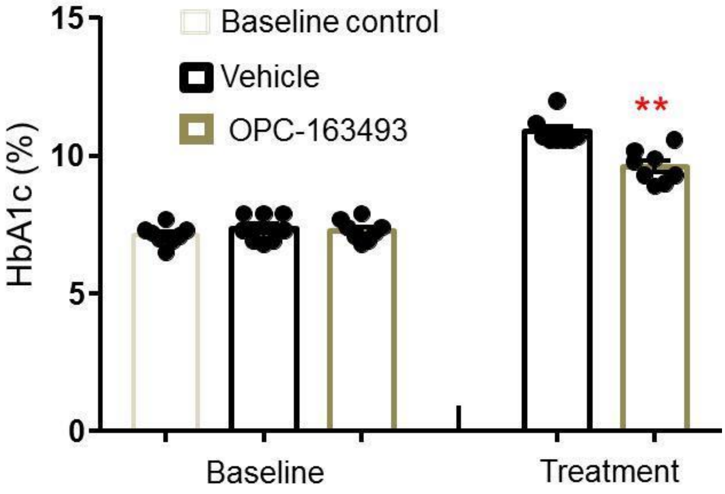

HbA1c (%)
15
10
5
0
Baseline control
Vehicle
OPC-163493
**
Baseline
Treatment

b

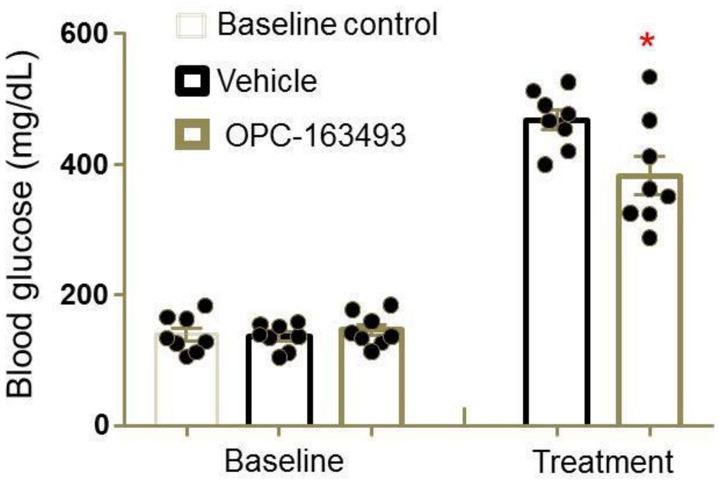

Blood glucose (mg/dL)
600
400
200
0
Baseline control
Vehicle
OPC-163493
*
Baseline
Treatment

c

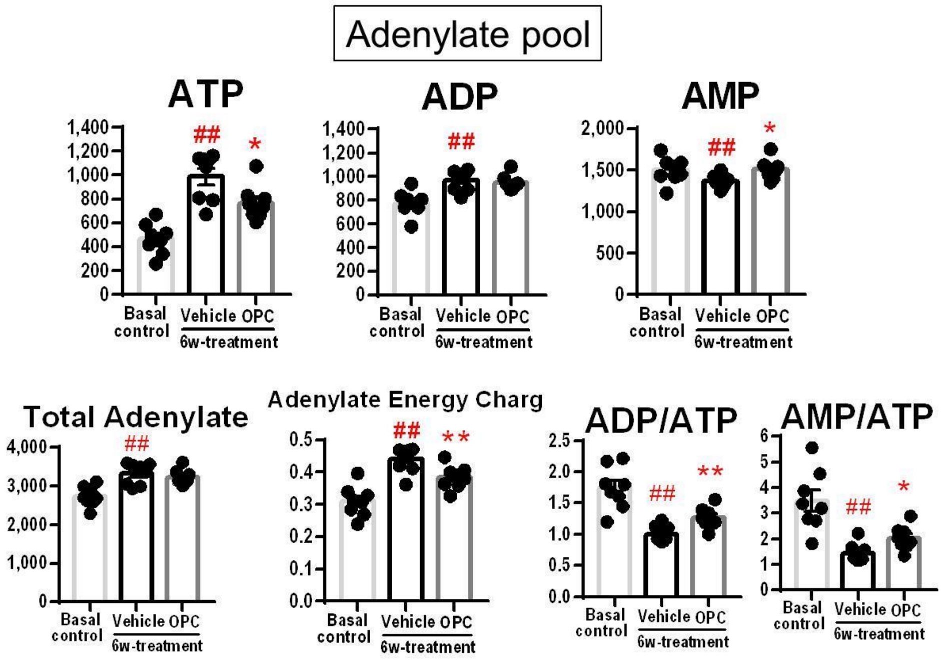

Adenylate pool
ATP
ADP
AMP
Total Adenylate
Adenylate Energy Charg
ADP/ATP
AMP/ATP
##
*
**
Basal control
Vehicle OPC
6w-treatment

d

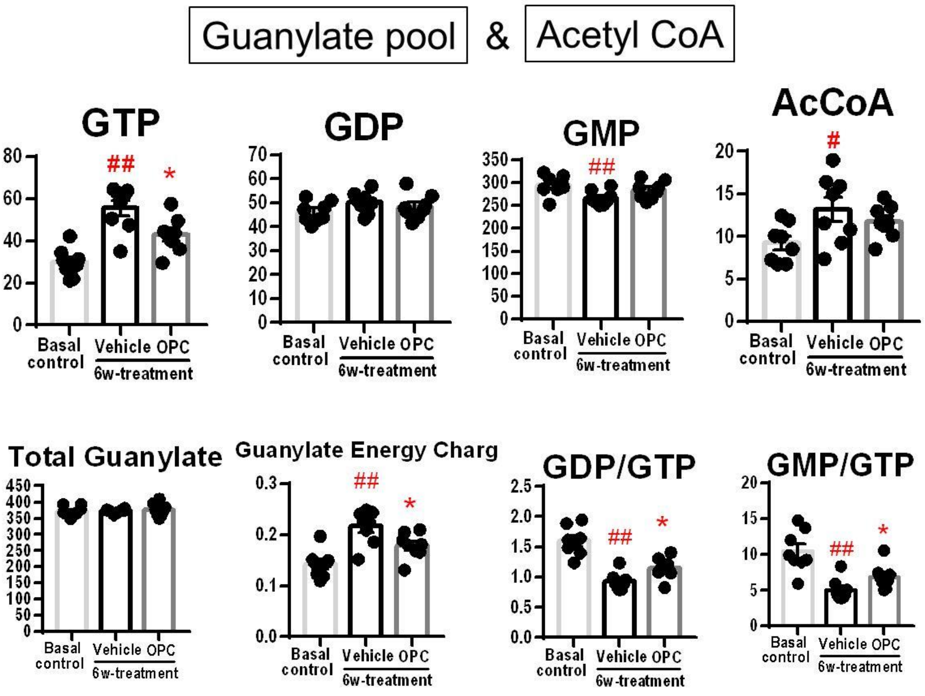

Guanylate pool
&
Acetyl CoA
GTP
GDP
GMP
AcCoA
Total Guanylate
Guanylate Energy Charg
GDP/GTP
GMP/GTP
##
#
*
Basal control
Vehicle OPC
6w-treatment

e

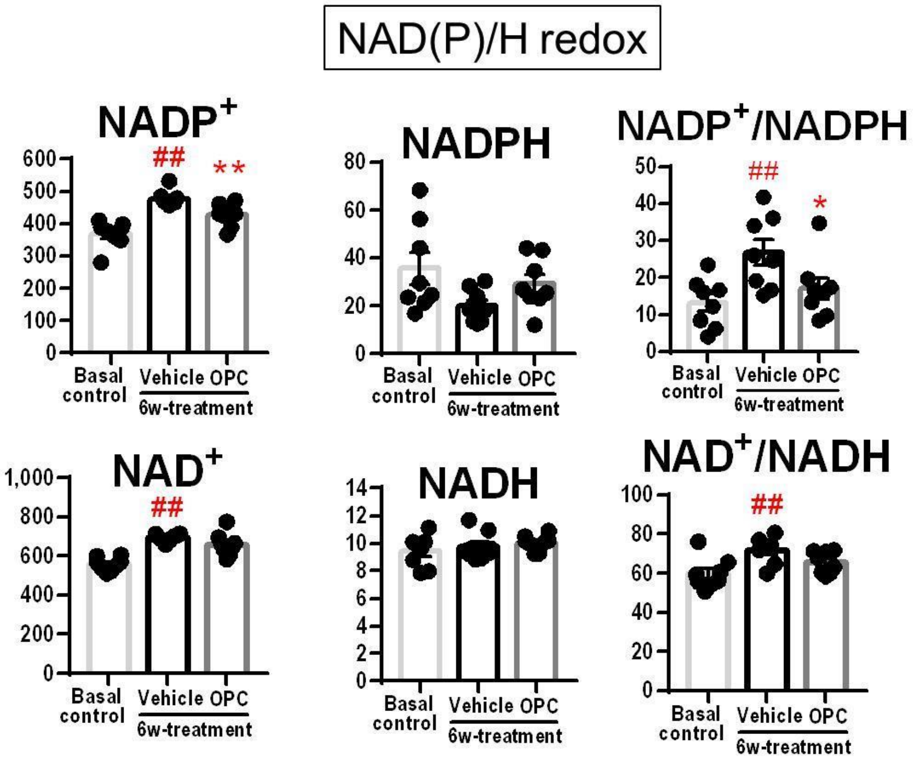

NAD(P)/H redox
NADP+
NADPH
NADP+/NADPH
NAD+
NADH
NAD+/NADH
##
**
*
Basal control
Vehicle
OPC
6w-treatment

f

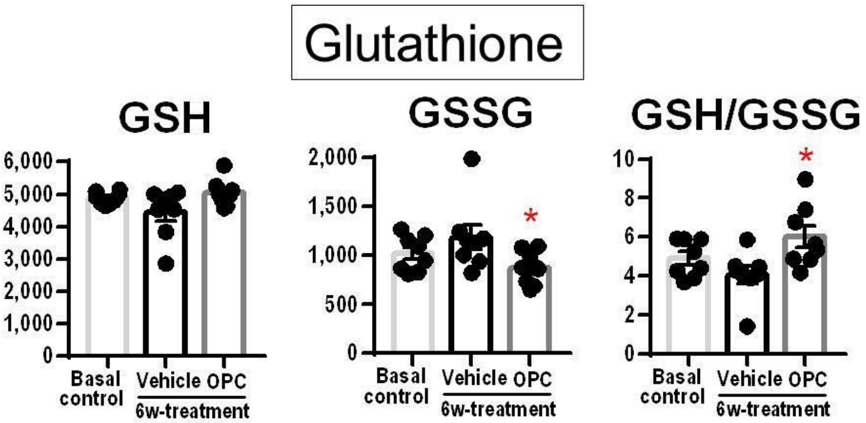

Glutathione
GSH
GSSG
GSH/GSSG
*
Basal control
Vehicle
OPC
6w-treatment

g

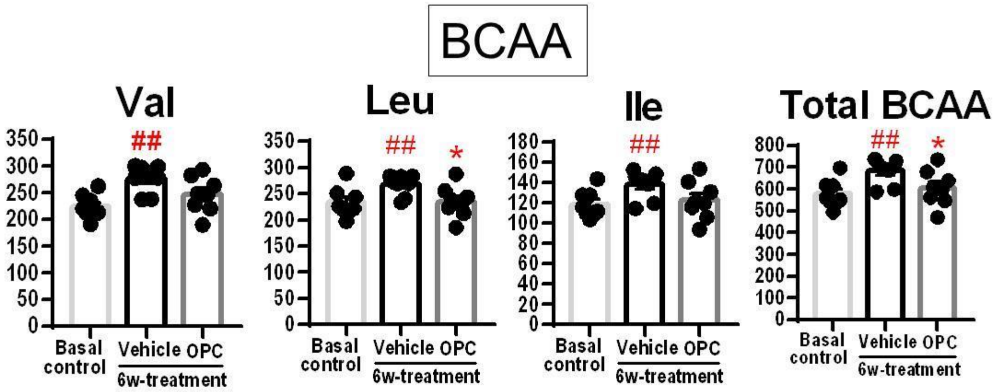

Supplementary Figure 5 Metabolomic analysis of in vivo effects of OPC-163493 in ZDF rats. (a) Effect of OPC-163493 on HbA1c value in ZDF rats. Significant efficacy was found in the OPC-163493-treated group (**P < 0.01, OPC vs Vehicle group by unpaired t-test). (b) Effect of OPC-163493 on fasting blood glucose level in ZDF rats. Significant efficacy was found in the OPC-163493-treated group (*P < 0.05, OPC vs Vehicle group by unpaired t-test). (c) Effects on adenylate pool. (d) Effects on guanylate pool and Acetyl CoA. (e) Effects on NAD(P)H redox. (f) Effects on glutathione redox. (g) Effects of BCAA metabolism. *P < 0.05, **P < 0.01, Vehicle group vs OPC-treatment group by unpaired t-test; #P < 0.05, ##P < 0.01, Baseline control group vs Vehicle group by unpaired t-test. All units of vertical lines in metabolite graphs are nmol g^{-1} of tissue (except ratios of substrates). Abbreviations and meanings of metabolic parameters are as in Supplementary Table 20.

a

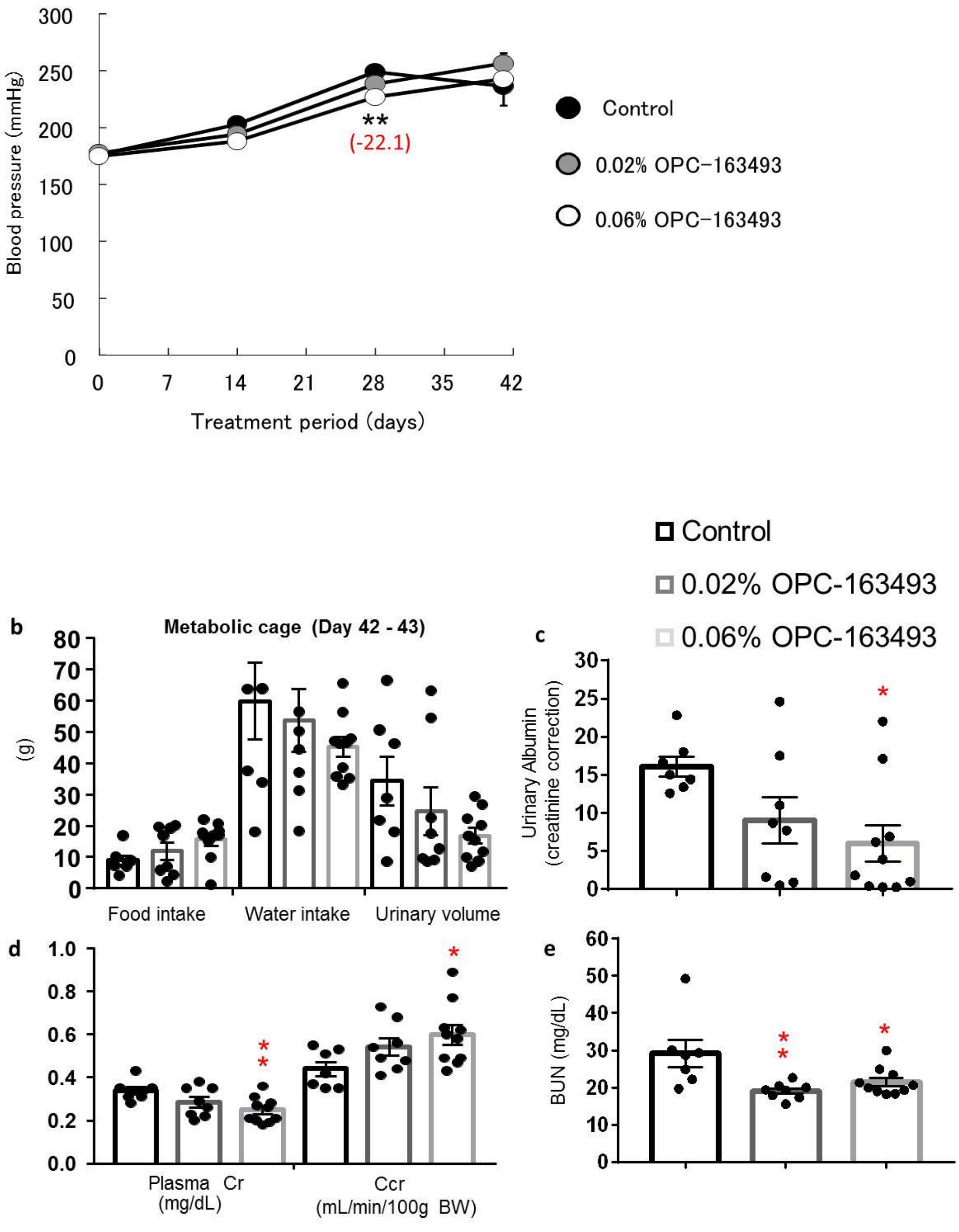

Supplementary Figure 6 Beneficial effects of OPC-163493 in salt-loaded SHRSPs. (a) Blood pressure-lowering effect. Systolic blood pressures (SBPs) are shown. Data represent mean ± SE (n = 10). As significant decreases were found in the overall treatment differences in SBP by the MMRM method (0.06% OPC vs Control chow group, P < 0.01), treatment-by-time interaction was then estimated by contrast-averaging the corresponding

treatment differences at each time point using the MMRM method (**P < 0.01). Since animal deaths were already seen in the control group and 0.02% mixed chow group by Day 41 (Supplementary Table 22), data on Day 41 were not included in the statistical analysis. (b) food intake, water drinking, urinary volume obtained from metabolic cages, (c) urinary albumin, (d) plasma creatinine (Cr) and Cr clearance (Ccr) (e) blood urea nitrogen (BUN) Data represent mean ± SE (Control: n = 7, 0.02% OPC: n = 8, 0.06% OPC: n = 10, * P < 0.05, ** P < 0.01 by Dunnett's test).

a

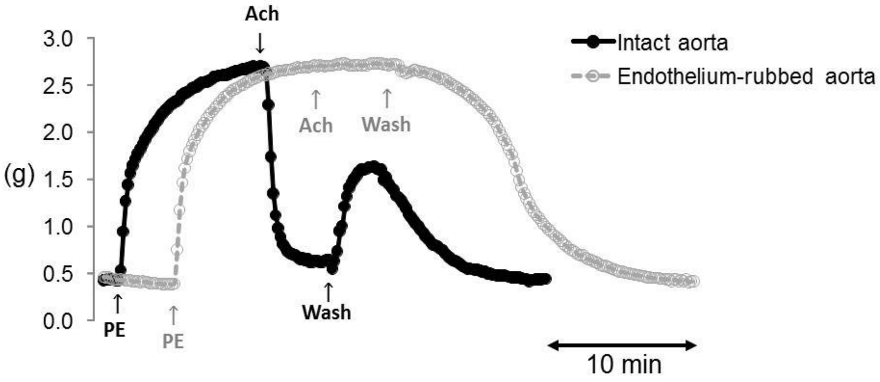
Intact aorta
Endothelium-rubbed aorta
(g)
3.0
2.5
2.0
1.5
1.0
0.5
0.0
Ach
Ach
Wash
Wash
PE
PE
10 min

b

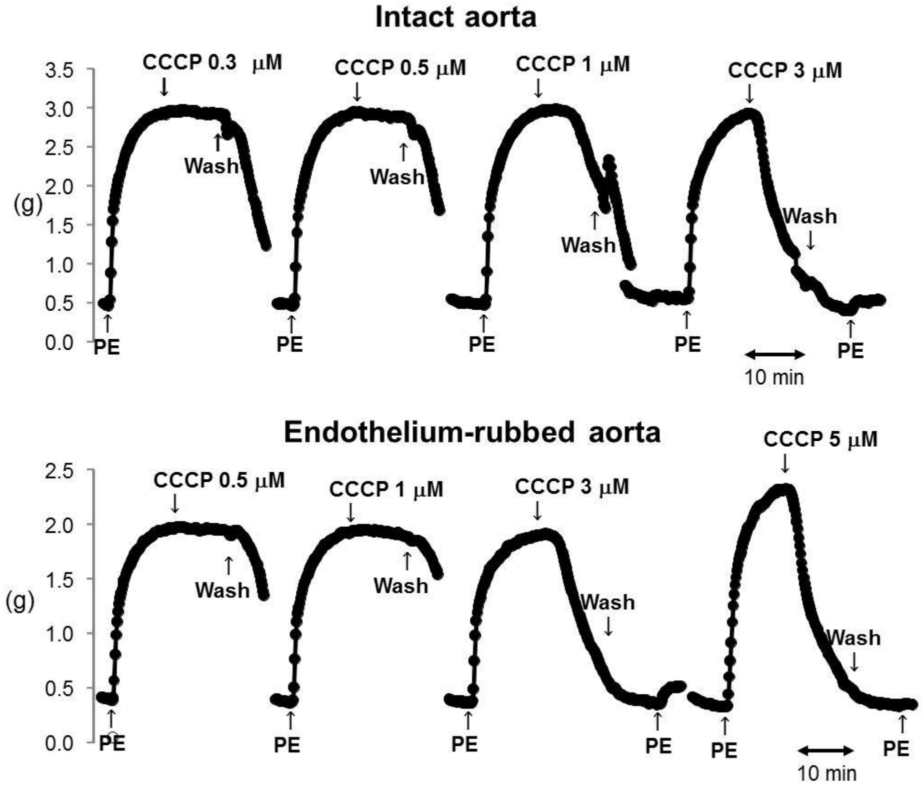
Intact aorta
CCCP 0.3 μM
CCCP 0.5 μM
CCCP 1 μM
CCCP 3 μM
Wash
Wash
Wash
Wash
(g)
3.5
3.0
2.5
2.0
1.5
1.0
0.5
0.0
PE
PE
PE
PE
PE
10 min
Endothelium-rubbed aorta
CCCP 0.5 μM
CCCP 1 μM
CCCP 3 μM
CCCP 5 μM
Wash
Wash
Wash
Wash
(g)
2.5
2.0
1.5
1.0
0.5
0.0
PE
PE
PE
PE
PE
PE
10 min

c

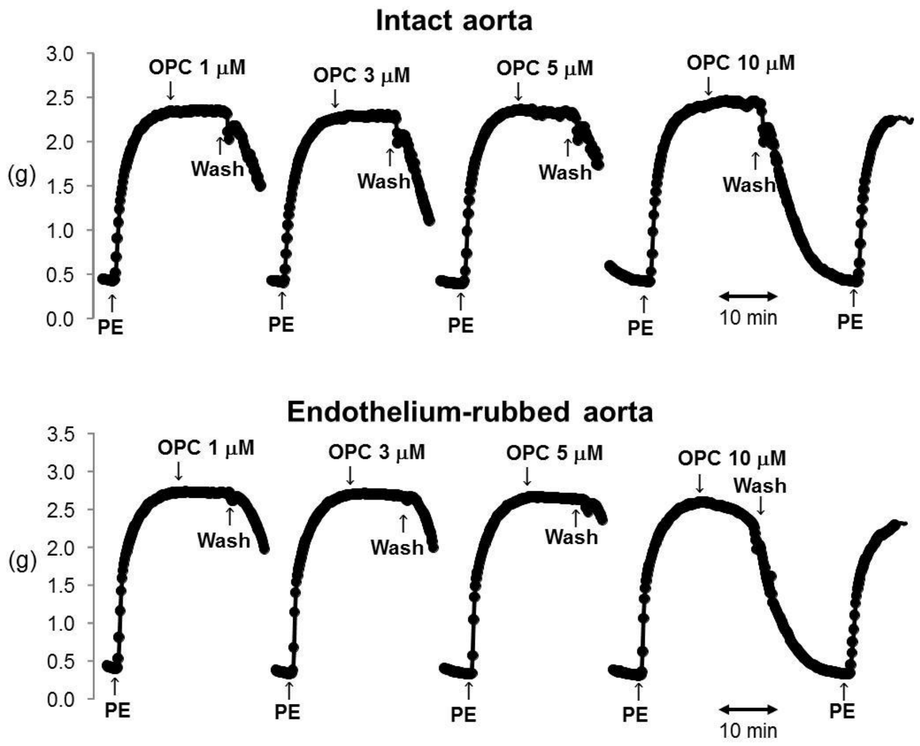

d

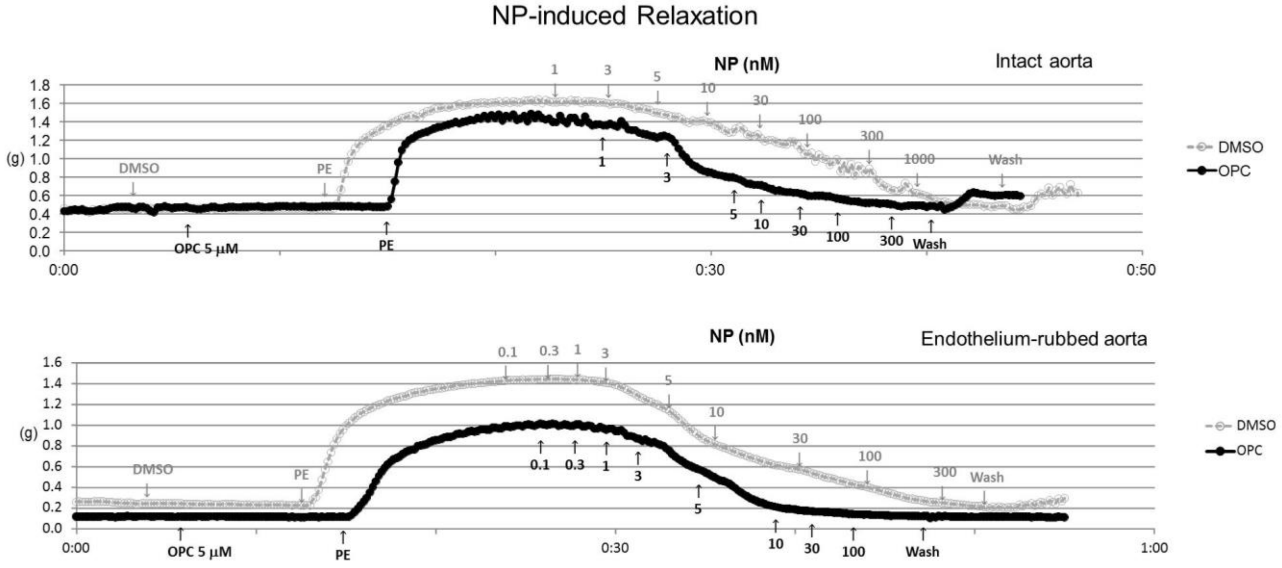

Supplementary Figure 7 Isometric tension recordings obtained from rat thoracic aortas. (a) Responses of intact and endothelium-rubbed aortas to PE (1 μM) and Ach (10 μM). The tension values were recorded at 1-sec intervals, and every 10 sec the average was calculated for graph-plotting. Representative graph is shown. (b) CCCP-induced relaxation

of rat aortas constricted by PE. Because the response gradually continued for a substantial duration, measurement was stopped at 10 min after CCCP addition. Representative graph is shown. (c) Effect of OPC-163493 on rat aortas constricted by PE. Representative graph is shown. (d) Sensitization of OPC-163493 to NP-induced relaxation of rat aortas. Representative graph is shown.

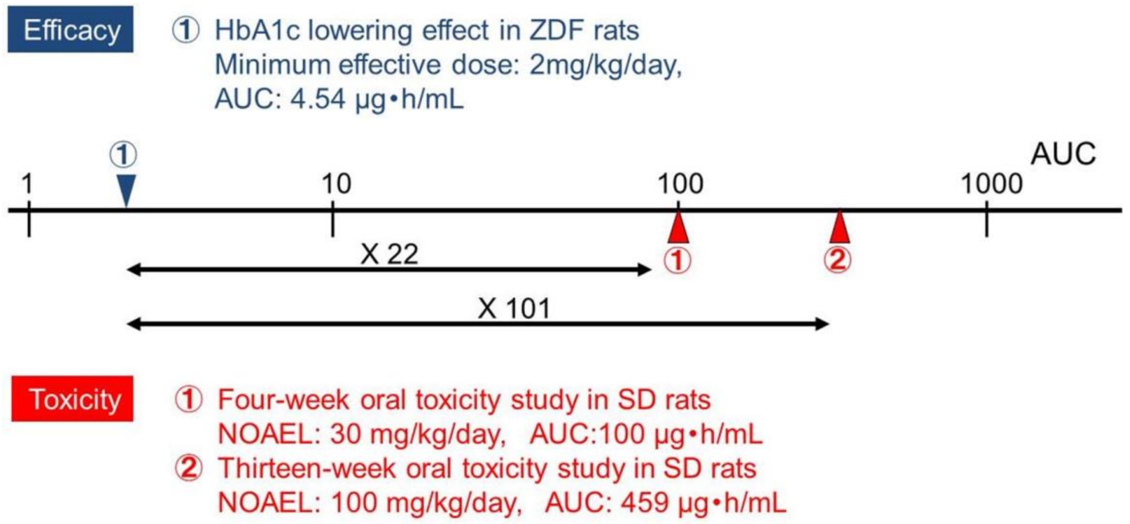

Supplementary Figure 8 Efficacy and toxicity of OPC-163493 in rats. Minimum effective dose was demonstrated in the efficacy study in ZDF rats (Fig. 2a and Supplementary Table 5).

a

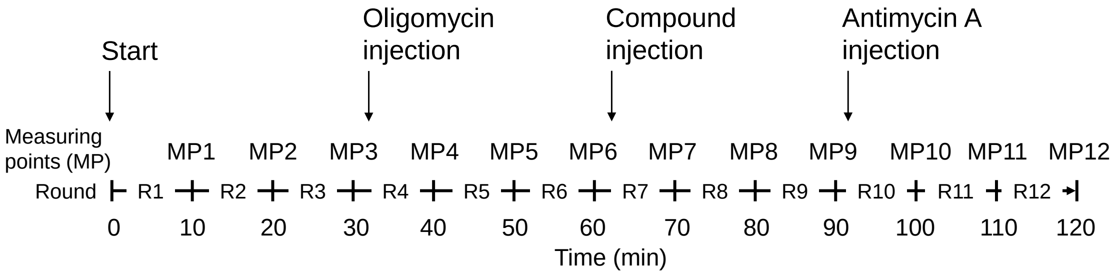

b

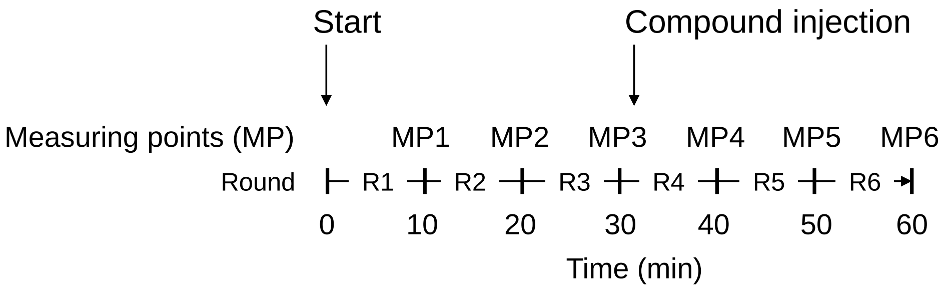

Supplementary Figure 9 Schemes of compound injections and OCR, CDER and ECAR measuring points. (a) Quantification of mUncoupling activity. (b) Measurements of cellular fluxes.

Supplementary Tables

Supplementary Table 1 Radioactivity in tissues after single oral administration of [^{14}C]-OPC-163493 suspension at 1 mg/kg to male rats by QWBA method

Tissue	Radioactivity Concentration (ng eq/g) (Tissue/Blood Ratio)				
	2 h	8 h	12 h	24 h	72 h
Blood	127.5 (1.0)	114.5 (1.0)	43.99 (1.0)	NC (NC)	NC (NC)
Cerebrum	BLQ (0.0)	BLQ (NC)	BLQ (NC)	BLQ (NC)	NC (NC)
Cerebellum	BLQ (0.0)	BLQ (NC)	BLQ (NC)	NC (NC)	NC (NC)
Medulla oblongata	BLQ (0.0)	BLQ (NC)	BLQ (NC)	NC (NC)	NC (NC)
Pituitary gland	60.50 (0.5)	36.73 (0.3)	30.29 (0.7)	NC (NC)	NC (NC)
Eyeball	BLQ (0.0)	BLQ (NC)	BLQ (NC)	NC (NC)	NC (NC)
Harderian gland	58.40 (0.5)	45.96 (0.4)	30.75 (0.7)	NC (NC)	NC (NC)
Submaxillary gland	109.4 (0.9)	91.37 (0.8)	48.18 (1.1)	NC (NC)	NC (NC)
Thyroid	30.13 (0.2)	28.38 (0.2)	49.83 (1.1)	NC (NC)	NC (NC)
Heart	137.5 (1.1)	112.9 (1.0)	44.32 (1.0)	NC (NC)	NC (NC)
Lung	179.2 (1.4)	168.7 (1.5)	46.42 (1.1)	NC (NC)	NC (NC)
Thymus	36.96 (0.3)	48.59 (0.4)	25.88 (0.6)	NC (NC)	NC (NC)
Skin	41.05 (0.3)	48.89 (0.4)	BLQ (NC)	70.40 (NC)	BLQ (NC)
Liver	916.1 (7.2)	806.0 (7.0)	762.7 (17.3)	175.6 (NC)	NC (NC)
Kidney (cortex)	1483 (11.6)	840.3 (7.3)	690.3 (15.7)	198.1 (NC)	NC (NC)
Kidney (medulla)	245.7 (1.9)	1588 (13.9)	122.3 (2.8)	21.99 (NC)	NC (NC)
Adrenal gland	285.5 (2.2)	125.2 (1.1)	52.38 (1.2)	BLQ (NC)	NC (NC)
Spleen	70.56 (0.6)	48.63 (0.4)	23.79 (0.5)	BLQ (NC)	NC (NC)
Pancreas	92.51 (0.7)	95.37 (0.8)	42.80 (1.0)	BLQ (NC)	NC (NC)
Muscle	NC (0.0)	36.45 (0.3)	BLQ (NC)	NC (NC)	NC (NC)
Brown fat	38.89 (0.3)	20.89 (0.2)	BLQ (NC)	NC (NC)	NC (NC)
Fat	BLQ (0.0)	BLQ (NC)	BLQ (NC)	BLQ (NC)	NC (NC)
Femur	NC (0.0)	BLQ (NC)	NC (NC)	NC (NC)	NC (NC)
Bone marrow	NC (0.0)	54.36 (0.5)	21.12 (0.5)	NC (NC)	NC (NC)
Testis	BLQ (0.0)	BLQ (NC)	BLQ (NC)	NC (NC)	NC (NC)
Seminal vesicle	25.72 (0.2)	BLQ (NC)	BLQ (NC)	NC (NC)	NC (NC)
Stomach	960.3 (7.5)	776.7 (6.8)	954.6 (21.7)	BLQ (NC)	NC (NC)
Small intestine	2254 (17.7)	1954 (17.1)	1285 (29.2)	778.4 (NC)	NC (NC)
Large intestine	34.03 (0.3)	23.57 (0.2)	487.8 (11.1)	3066 (NC)	NC (NC)

BLQ: Below lower limit of quantification (20 ng eq g^{-1}). NC: not evaluated because of the low radioactivity in the case of radioactivity concentration and not calculated in the case of tissue/blood ratio. A linear calibration curve was obtained in the range of 20−20,000 ng eq mL^{-1}.

Supplementary Table 2 Radioactivity in tissues after single oral administration of [^{14}C]-OPC-163493 suspension at 1 mg/kg to Day 17 pregnancy rats by QWBA method

Tissue	Radioactivity Concentration (ng eq/g) (Tissue/Blood Ratio)				
	2 h	8 h	12 h	24 h	72 h
Blood	306.0 (1.0)	366.2 (1.0)	64.08 (1.0)	NC (NC)	NC (NC)
Cerebrum	BLQ (NC)	BLQ (NC)	BLQ (NC)	NC (NC)	NC (NC)
Cerebellum	BLQ (NC)	BLQ (NC)	BLQ (NC)	NC (NC)	NC (NC)
Medulla oblongata	BLQ (NC)	BLQ (NC)	NC (NC)	NC (NC)	NC (NC)
Pituitary gland	BLQ (NC)	219.8 (0.6)	NC (NC)	NC (NC)	NC (NC)
Eyeball	BLQ (NC)	BLQ (NC)	BLQ (NC)	NC (NC)	NC (NC)
Harderian gland	163.6 (0.5)	209.0 (0.6)	30.46 (0.5)	NC (NC)	NC (NC)
Submaxillary gland	243.2 (0.8)	307.6 (0.8)	61.08 (1.0)	NC (NC)	NC (NC)
Thyroid	94.28 (0.3)	172.5 (0.5)	NC (NC)	NC (NC)	NC (NC)
Heart	228.3 (0.7)	369.5 (1.0)	65.56 (1.0)	NC (NC)	NC (NC)
Lung	368.5 (1.2)	409.3 (1.1)	108.8 (1.7)	NC (NC)	NC (NC)
Thymus	116.3 (0.4)	187.4 (0.5)	40.07 (0.6)	NC (NC)	NC (NC)
Skin	53.55 (0.2)	147.1 (0.4)	27.58 (0.4)	BLQ (NC)	NC (NC)
Liver	1590 (5.2)	1816 (5.0)	811.5 (12.7)	233.8 (NC)	NC (NC)
Kidney (cortex)	1114 (3.6)	1409 (3.8)	467.6 (7.3)	64.04 (NC)	NC (NC)
Kidney (medulla)	340.7 (1.1)	3387 (9.2)	154.6 (2.4)	312.1 (NC)	NC (NC)
Adrenal gland	350.1 (1.1)	379.2 (1.0)	86.31 (1.3)	NC (NC)	NC (NC)
Spleen	137.0 (0.4)	224.6 (0.6)	45.85 (0.7)	NC (NC)	NC (NC)
Pancreas	186.3 (0.6)	378.1 (1.0)	25.22 (0.4)	NC (NC)	NC (NC)
Muscle	63.48 (0.2)	96.12 (0.3)	22.03 (0.3)	NC (NC)	NC (NC)
Brown fat	160.1 (0.5)	113.3 (0.3)	NC (NC)	NC (NC)	NC (NC)
Fat	BLQ (NC)	23.37 (0.1)	BLQ (NC)	BLQ (NC)	NC (NC)
Femur	BLQ (NC)	BLQ (NC)	BLQ (NC)	NC (NC)	NC (NC)
Bone marrow	98.24 (0.3)	176.3 (0.5)	31.12 (0.5)	NC (NC)	NC (NC)
Mammary gland	80.92 (0.3)	93.63 (0.3)	BLQ (NC)	NC (NC)	NC (NC)
Placenta	80.65 (0.3)	213.1 (0.6)	46.44 (0.7)	BLQ (NC)	NC (NC)
Amniotic fluid	BLQ (NC)	BLQ (NC)	BLQ (NC)	BLQ (NC)	NC (NC)
Fetus	BLQ (NC)	BLQ (NC)	BLQ (NC)	BLQ (NC)	NC (NC)
Uterus	104.1 (0.3)	526.9 (1.4)	441.0 (6.9)	542.4 (NC)	NC (NC)
Ovary	85.73 (0.3)	340.4 (0.9)	32.41 (0.5)	NC (NC)	NC (NC)
Stomach	167.9 (0.5)	309.7 (0.8)	376.5 (5.9)	217.5 (NC)	NC (NC)
Small intestine	356.8 (1.2)	1909 (5.2)	1257 (19.6)	699.5 (NC)	NC (NC)
Large intestine	166.1 (0.5)	197.8 (0.5)	655.1 (10.2)	411.4 (NC)	NC (NC)

BLQ: Below lower limit of quantification (20 ng eq g^{-1}). NC: not evaluated because of the low radioactivity in the case of radioactivity concentration and not calculated in the case of tissue/blood ratio. A linear calibration curve was obtained in the range of 20−20,000 ng eq mL^{-1}.

Supplementary Table 3 Pharmacokinetic parameters of radioactivity in the plasma and liver following single oral administration of 1 mg/kg 14C-OPC-163493 in fasted male rats

Parameter	Plasma	Liver
C_{max} (μg eq/mL or g)	0.5371	2.746
t_{max} (h)	2.0	4.0
AUC_{24h} (μg eq・h/mL or g)	5.058	31.82

Supplementary Table 4 HbA1c baseline of ZDF rats at the age of 11 weeks

Group	Baseline (%)
Vehicle	6.24 ± 0.08
1 mg/kg/day	6.25 ± 0.09
2 mg/kg/day	6.33 ± 0.09
4 mg/kg/day	6.26 ± 0.11
10 mg/kg/day	6.33 ± 0.09

Data represent mean ± SE (n = 16).

Supplementary Table 5 Pharmacokinetic parameters of OPC-163493 in ZDF rats

Daily dose (mg/kg/day)	Single dose (mg/kg)	C_{max} (μg/mL)	t_{max} (h)	AUC_t (μg·h/mL)
1	0.5	0.2948 ± 0.0319	11.3 ± 0.7	2.925 ± 0.363
2	1	0.4450 ± 0.0242	10.0 ± 1.0	4.540 ± 0.284
4	2	1.193 ± 0.214	9.7 ± 1.2	9.563 ± 1.258
10	5	4.082 ± 0.773	8.8 ± 0.2	34.16 ± 2.99

Data represent mean ± SE (n = 3). OPC-163493 suspensions were administered twice at 0 h (9AM) and 8 h (5PM).

Supplementary Table 6 HbA1c baseline of Akita mice at the age of 6 weeks

Group	Baseline (%)
Control	7.29 ± 0.16
0.005%	7.18 ± 0.20
0.01%	7.14 ± 0.18
0.02%	7.18 ± 0.19

Data represent mean ± SE (n = 12).

Supplementary Table 7 The Diurnal change of plasma OPC-163493 concentrations in Akita mice following 6-week dietary administration

Group		6 AM	12 noon	6 PM	12 midnight
0.005 % OPC-163493	mean	0.7726	0.6624	0.2517	0.8378
	SE	0.0592	0.0916	0.0315	0.0428
0.01% OPC-163493	mean	1.634	0.9069	0.6717	1.647
	SE	0.168	0.0707	0.2254	0.288
0.02% OPC-163493	mean	4.246	2.384	1.840	4.064
	SE	0.155	0.208	0.260	0.633

Mean and SE in each group at each time point are represented in the table (µg mL^{-1}, n=3).

Supplementary Table 8 HbA1c baseline of ZDF rats at the age of 27 weeks

Group	Baseline (%)
Vehicle	10.28 ± 0.27
2 mg/kg/day	10.25 ± 0.06
6 mg/kg/day	10.28 ± 0.20
10 mg/kg/day	10.27 ± 0.22

Data represent mean ± SE (n = 6).

Supplementary Table 9 Pharmacokinetic parameters of OPC-163493 in old ZDF rats						
Compound	n	Daily dose (mg/kg/day)	Single dose (mg/kg)	C_{max} (µg/mL)	T_{max} (h)	AUC_{0-24} (µg・h/mL)
OPC-163493	3	2	1	0.467 ±0.071	9.0 ± 0.0	6.05 ±0.89
	3	6	3	1.538 ± 0.256	9.5 ± 0.3	16.60 ±1.81
	2	10	5	2.268 ± 0.353	9.0 ±0.0	18.57 ±4.30

Data represent mean ± SE. OPC-163493 suspensions were administered twice at 0 h (9AM) and 8 h (5PM).

Supplementary Table 10 Plasma OPC-163493 concentrations at 6 AM in OLETF rats following 1-week dietary administration		
Group		6 AM
0.01 % OPC-163493	mean	0.271
	SE	0.006
0.02% OPC-163493	mean	0.607
	SE	0.051
0.06% OPC-163493	mean	2.112
	SE	0.045

Mean and SE at 6AM are represented in the table (µg mL^{-1}, n=3).

Supplementary Table 11 The diurnal change of plasma OPC-163493 concentrations in ZDF(M) rats following 4-week dietary administration					
Group		6 AM	12 noon	6 PM	12 midnight
0.04 % OPC-163493	mean	4.536	4.131	3.938	4.064
	SE	0.748	0.259	0.448	0.436

Mean and SE at each time point are represented in the table (µg mL^{-1}, n=3).

Supplementary Table 12 HbA1c baseline of ob/ob mice at the age of 8 weeks

Group	n	Baseline (%)
Control	7	4.89 ± 0.10
0.005%	8	4.95 ± 0.11
0.01%	8	4.95 ± 0.10
0.02%	8	4.91 ± 0.12

Data represent mean ± SE.

Supplementary Table 13 Plasma OPC-163493 concentrations at 6 AM in ob/ob mice following 10-week dietary administration

Group		6 AM
0.005 % OPC-163493	mean	0.322
	SE	0.013
0.01% OPC-163493	mean	0.990
	SE	0.119
0.02% OPC-163493	mean	1.962
	SE	0.135

Mean and SE at 6AM are represented in the table (µg mL^{-1}, n=3).

Supplementary Table 14: Please see Source Data file worksheet “sTable 14”.

Supplementary Table 15 Pharmacokinetic parameters of OPC-163493 in HDF SD rats

Compound	n	Daily dose (mg/kg/day)	Single dose (mg/kg)	C_{max} (µg/mL)	T_{max} (h)	AUC_{0-24} (µg・h/mL)
OPC-163493	3	2	1	0.640 ±0.139	10.7 ± 0.7	7.24 ±1.31
	3	4	2	1.296 ± 0.081	8.0 ± 3.1	17.19 ±0.97

Data represent mean ± SE. OPC-163493 suspensions were administered twice at 0 h (9 AM) and 8 h (5 PM).

Supplementary Table 16 Long-term effect of OPC-163493 dosing with mixed chow on HbA1c values in male OLETF rats

Group	HbA1c (%)						
	0w	2w	4w	8w	13w	16w	20w
Contro	5.41	5.22	5.28	5.86	5.85	6.03	6.06
0.01%	5.47	5.15	5.24	5.85	5.81	5.81	5.80
0.02%	5.45	4.99	4.88*	5.53*	5.45*	5.62*	5.55**
0.06%	5.36	4.85*	4.65**	5.22**	5.19**	5.18**	5.24**
LETO							3.26##

Data represent mean (n = 14) except for LETO (n = 6). Significant efficacy was found in the 0.02% and 0.06% OPC-mixed chow-treated groups using a mixed model for repeated measures (MMRM) method followed by Dunnett's test (OPC vs Control chow group, $P < 0.01$). Treatment-by-time interaction was then estimated by contrast-averaging the corresponding treatment differences at each time point using the MMRM method (*$P < 0.05$, **$P < 0.01$). A significant difference was also observed between the LETO rat group and the OLETF rat (control chow) group using an unpaired t-test (##$P < 0.01$).

SupplementaryTable 17 Effects of OPC-163493 on hyperinsulinemic-euglycemic clamp parameters

State	Parameter	Group	Mean ± SE (n = 12)	Difference (OPC - control)	P
			(mg/kg/min)		(Unpaired t-test)
Basal (-20 - 0 min)	HGP	Control	8.35 ± 0.75	-2.52	P < 0.05
		OPC-163493	5.83 ± 0.58		
Clamp (60 - 120 min)	GIR	Control	12.47 ± 1.30	1.33	Not significant
		OPC-163493	13.80 ± 1.41		
	HGP	Control	-8.36 ± 1.36	-1.52	Not significant
		OPC-163493	-9.88 ± 1.48		
	Rd	Control	3.91 ± 0.08	-0.31	P < 0.05
		OPC-163493	3.60 ± 0.10		

Supplementary Table 18: Please see Source data file worksheet “sTable 18”.

Supplementary Table 19 Pharmacokinetic parameters of OPC-163493 in ZDF rats

Daily dose (mg/kg/day)	Single dose (mg/kg)	C_{max} (μg/mL)	t_{max} (h)	AUC_t (μg·h/mL)
6	3	1.122 ±0.165	9.0 ±0.0	10.64 ±0.76

Data represent mean ± SE (n = 3). OPC-163493 suspensions were administered twice at 0 h (9 AM) and 8 h (5 PM). Reasonable PK parameters were obtained once more and should be compared with the previous study (Supplementary Table 5).

Supplementary Table 20: Please see Source Data file worksheet “sTable 20”.

Supplementary Table 21 Parameters of OPC-163493 intervention study in salt-loaded SHRSPs (1st exp.)

Group	Beginning No. of animals		BW (g)				SBP (mmHg)			
	n		Baseline	Day 5	Day 26	Day 40	Baseline	Day 5	Day 27	Day 41
Control	16	Mean	139.5	179.9	256.1	272.8	174.8	174.0	242.5	260.5
		SE	1.3	1.6	2.2	4.7	2.4	2.8	2.6	4.1
0.06% OPC-163493	16	Mean	139.4	175.6	248.8	272.0	174.8	169.0	228.3$^{\$\$}$	243.5$^{\$\$}$
		SE	1.5	1.6	2.7	3.5	2.1	3.3	3.0	4.3

Group		Madian days to stroke symptoms	Madian days to deaths		Metabolic cage between Day 48 and 49 (24h)					Plasma OPC conc. at 6AM on Day 51	
					n	Food intake (g)	Water drinking (g)	Urinary volume (g)	Urinary albumin /creatinine	n	(μg/mL)
Control	Median	47.5	54.0	Mean	11	5.9	60.2	47.0	14.3		
	95% CI	43.0-48.0	50.0-58.0	SE		2.1	5.4	4.2	1.6		
0.06% OPC-163493	Median	55.5$^{\#\#}$	63.5$^{\#\#}$	Mean	15	16.5**	57.8	39.3	4.6**	7	2.48
	95% CI	52.0-56.0	60.0-64.0	SE		1.1	1.9	2.1	1.1		0.29

SBP: systolic blood pressure. A significant decrease was found in the overall treatment differences in SBP using the MMRM method (OPC vs Control chow group, P < 0.01); treatment-by-time interaction was then estimated by contrast averaging the corresponding treatment differences at each time point using the MMRM method ($^{\$\$}$ P < 0.01). $^{\#\#}$ p<0.01, OPC vs Control group (Log-Rank test). ** p<0.01, OPC vs Control group (unpaired t-test).

Supplementary Table 22 Parameters of OPC-163493 intervention study in salt-loaded SHRSPs (2nd exp.)

Group	Beginning No. of animals	Remaining No. of animals					BW (g)							
	n	Day 33	Day 35	Day 38	Day 41		Baseline	Day 5	Day 12	Day 19	Day 26	Day 33	Day 42	Day 43
Control	10	9	8	7	7	Mean	131.6	179.0	215.4	236.1	251.8	254.4	244.5	237.2
						SE	4.3	2.6	2.8	3.3	4.0	9.9	13.9	11.7
0.02% OPC-163493	10	10	9	9	8	Mean	131.6	172.4	205.9	230.2	248.3	256.5	253.8	249.7
						SE	3.0	2.8	5.9	5.5	5.5	6.6	10.1	10.8
0.06% OPC-163493	10	10	10	10	10	Mean	131.4	176.2	208.5	230.2	245.3	257.2	254.8	252.7
						SE	5.5	3.7	3.7	2.8	3.0	3.2	6.9	7.9

Group		Food intake (g)								Water drnking (g)																
		Day 9	Day 16	Day 19	Day 26	Day 30	Day 33	Day 37	Day 42	Day 2	Day 5	Day 9	Day 12	Day 16	Day 19	Day 23	Day 26	Day 28	Day 30	Day 33	Day 35	Day 37	Day 39	Day 41	Day 42	
Control	Mean	16.8	18.5	19.2	18.8	17.3	16.4	16.5	12.4	35.4	36.7	40.5	43.8	48.1	51.0	48.1	53.1	57.9	50.9	58.5	61.2	59.6	61.6	78.7	67.6	
	SE	0.2	0.3	0.3	0.4	0.8	1.8	1.9	1.8	1.4	1.6	1.6	1.6	2.5	2.8	2.2	2.4	2.4	2.9	6.0	4.2	7.7	8.3	6.4	6.8	
0.02% OPC-163493	Mean	15.8	18.2	19.3	19.6	19.3	18.6	18.2	15.7	35.6	36.2	36.9	40.7	42.3	45.1	42.3	45.4	51.6	45.3	49.0	46.7	45.2	46.3	51.0	48.0	##
	SE	0.3	0.4	0.4	0.5	0.5	1.4	1.0	1.6	1.6	1.6	1.5	1.8	2.2	2.5	2.3	2.3	2.8	3.1	1.8	3.5	7.7	4.4	5.4	8.3	
0.06% OPC-163493	Mean	16.1	18.1	19.4	19.6	19.3	19.5	18.1	16.9	34.2	35.5	35.4	37.7	38.5	42.8	37.7	49.5	51.2	43.3	54.1	51.2	45.6	57.1	56.9	45.6	#
	SE	0.3	0.2	0.2	0.3	0.3	0.4	0.8	1.4	1.7	1.7	1.3	1.1	1.5	1.4	4.1	3.1	2.7	1.8	3.2	2.9	7.4	2.4	3.9	3.7	

Group		SBP (mmHg)				Metabolic cage between Day 43 and 43 (24h)				Renal functions				Plasma OPC conc. at 6AM on Day 35	
		Base-line	Day 14	Day 28	Day 41	n	Food intake (g)	Water drinking (g)	Urinary volume (g)	Urinary albumin /creatinine	Plasma creatinine (mg/dL)	Creatinine clearance (mL/min/100g BW)	BUN (mg/dL)	n	(μg/mL)
Control	Mean	176.4	202.9	248.6	236.3	7	9.1	59.9	34.4	16.1	0.34	0.44	29.2		
	SE	5.2	4.0	6.4	17.4		1.5	12.4	7.8	1.3	0.02	0.03	3.7		
0.02% OPC-163493	Mean	177.4	194.0	238.3	256.2	8	11.9	53.7	24.8	9.1	0.28	0.54	19.1**	5	0.67
	SE	6.5	3.9	4.6	9.0		2.7	10.1	7.7	3.0	0.02	0.04	0.8		0.05
0.06% OPC-163493	Mean	174.7	187.9	226.5$$ ##	242.4	10	15.6	45.3	16.8	6.0*	0.25**	0.60*	21.5*	5	3.14
	SE	5.8	3.2	4.7	9.3		1.9	3.2	2.4	2.4	0.02	0.05	1.2		0.16

# p<0.05, ## p<0.01, OPC vs Control group (using the MMRM method followed by Dunnett's test).

$$ p<0.01, OPC vs Control group (comparison of treatment-by-time interaction at each time point using the post-hoc test of the MMRM method). * p<0.05, ** p<0.01, OPC vs Control group (Dunnett's test).

Supplementary Table 23 Sensitization of OPC-163493 to NP-induced relaxation of rat aortas

Intact aorta (n = 10)

	EC_{50}	L95%CL (nmol/L)	U95%CL	-fold
DMSO	36.14	23.60	55.34	6.59
OPC	5.487	3.106	9.691	

Endothelium-rubbed aorta (n = 10)

	EC_{50}	L95%CL (nmol/L)	U95%CL	-fold
DMSO	4.318	3.240	5.755	3.22
OPC	1.342	0.988	1.821	

EC_{50} values were estimated by regression analysis (NP concentrations were subjected to Log transformation).

Supplementary Table 24 Summary of 4-week repeated oral dosing toxicity study in rats

Dose（mg/kg）	30	100		300	
Sex and Number of Animals	M:10<br>F: 10	F:10	M: 10	F: 15	M:15
Death	-	-	-	4/15	-
General Condition, Body Weight	-	-	-	Salivation,<br>Body weight gain↓	Salivation,<br>Body weight gain↓<br>Body temperature↑
Hematology	-	-	Hb,<br>Ht, ↑	RET ↑<br>Eos↓	Hb, Ht, RBC ↑<br>Neut, Eos↓
Blood biochemistry	-	-	-	ALT, ALP, TBI, CHO, PL, TG↑	AST, ALT, ALP, TBI, CHO, PL, BUN ↑
Histopathological Change	-	-	Liver: Hypertrophy of hepatocyte		
			-	Liver: Granular eosinophilic cytoplasm<br>Pancreas: Islet cell necrosis	
				Kidney: Hyperplasia (transitional cells & urinary bladder)	Liver: Focal Necrosis
TK parameters:Day28<br>Cmax (mg/mL)<br>Tmax (h)<br>AUC24h (mg*h/mL)	M<br>12.0<br>2<br>100<br>F<br>16.8<br>2<br>95	49.4<br>2<br>398	33.5<br>2<br>369	91.8<br>2<br>1183	91.7<br>4<br>1400

Supplementary Table 25 Summary of 13-week repeated oral dosing toxicity study in rats

Dose（mg/kg）	10	30	100
Sex and Number of Animals	M:10<br>F: 10	M: 10<br>F: 10	M: 10<br>F: 10
Death	-	-	-
General Condition	-	-	-
Hematology	-	-	-
Blood biochemistry	-	-	-
Histopathological change	-	-	-
TK parameters: Day91<br>Cmax (mg/mL)<br>Tmax (h)<br>AUC24h (mg*h/mL)	M<br>4.7<br>1<br>43<br>F<br>6.6<br>2<br>50	M<br>15.9<br>2<br>144<br>F<br>20.1<br>2<br>164	M<br>36.2<br>4<br>459<br>F<br>56.2<br>1<br>477

No toxicological change was observed.

Supplementary Table 26 Toxic changes observed with a four-week, repeated oral dosing toxicity study in rats.

Body temperature (rectal temperature)

Sex: Male Unit: ˚C (Mean ± SD)

Dose (mg/kg) Stage	Number of Animals	Time	0 (control)	30	100	300
Week 2	5	Pre	37.30 ± 0.30	37.48 ± 0.22	37.28 ± 0.34	36.38 ± 0.22**
		4h	36.62 ± 0.33	36.60 ± 0.39	36.78 ± 0.30	37.76 ± 0.63**
Week 4	5	Pre	36.64 ± 0.35	36.72 ± 0.70	36.22 ± 0.74	36.50 ± 0.27
		4h	35.98 ± 0.34	35.84 ± 0.42	36.00 ± 0.32	36.68 ± 0.28**

*P<0.05, **P<0.01 vs control group.
When significant (P<0.05) difference was found in one-way ANOVA, Dunnett's test was followed for comparisons between the vehicle control and treated groups.

Body weight gain (Base: Day 1)

Sex: Male Unit: g

Dose (mg/kg)	0 (control)	30	100	300
Number of Animals	15	10	10	15
	Mean ± SD	Mean ± SD	Mean ± SD	Mean ± SD
Day	n	n	n	n
8	53.5 ± 10.2	54.8 ± 8.1	53.0 ± 9.4	39.5 ± 11.4**
	15	10	10	15
15	100.7 ± 21.5	99.9 ± 13.6	96.5 ± 17.7	76.9 ± 21.5**
	15	10	10	15
22	141.4 ± 31.8	141.6 ± 16.7	135.8 ± 26.2	105.6 ± 29.0**
	15	10	10	15
28	168.4 ± 38.3	168.0 ± 20.6	159.8 ± 33.1	118.6 ± 31.2**
	15	10	10	15

Sex: Female Unit: g

Dose (mg/kg)	0 (control)	30	100	300
Number of Animals	15	10	10	15
	Mean ± SD	Mean ± SD	Mean ± SD	Mean ± SD
Day	n	n	n	n
8	22.2 ± 5.5	20.8 ± 5.2	17.5 ± 4.5	19.5 ± 5.4
	15	10	10	15
15	39.7 ± 6.7	37.7 ± 8.5	34.8 ± 5.9	32.2 ± 9.5
	15	10	10	15
22	55.2 ± 11.2	54.1 ± 10.6	53.4 ± 10.4	31.8 ± 35.5*
	15	10	10	15
28	66.6 ± 12.4	64.0 ± 11.4	60.7 ± 12.1	54.6 ± 13.4
	15	10	10	12

*P<0.05, **P<0.01 vs control group.
When significant (P<0.05) difference was found in one-way ANOVA, Dunnett's test was followed for comparisons between the vehicle control and treated groups.

Hematology

Sex: Male Stage: Week 4

Dose (mg/kg)	0 (control)	30	100	300
Number of Animals	10	10	10	10
	Mean ± SD	Mean ± SD	Mean ± SD	Mean ± SD
Item (unit)	n	n	n	n
Hb (g/dL)	15.10 ± 0.34	15.27 ± 0.70	15.99 ± 0.68**	16.50 ± 0.71**
	10	10	10	9
Ht (%)	45.11 ± 1.19	45.98 ± 2.03	47.16 ± 1.90*	49.03 ± 2.08**
	10	10	10	9
RBC ($x10^6/mm^3$)	7.714 ± 0.277	7.772 ± 0.314	7.954 ± 0.290	8.294 ± 0.383**
	10	10	10	9
Neut ($x10^3/mm^3$)	1.790 ± 0.439	1.562 ± 0.482	1.293 ± 0.286*	1.337 ± 0.326*
	10	10	10	9
Eos ($x10^3/mm^3$)	0.093 ± 0.018	0.095 ± 0.035	0.093 ± 0.037	0.053 ± 0.014*
	10	10	10	9

Sex: Female Stage: Week 4

Dose (mg/kg)	0 (control)	30	100	300
Number of Animals	10	10	10	15
	Mean ± SD	Mean ± SD	Mean ± SD	Mean ± SD
Item (unit)	n	n	n	n
RET ($x10^4/mm^3$)	15.478 ± 3.581	17.239 ± 3.946	17.876 ± 4.066	27.253 ± 7.421**
	10	10	10	7
Eos ($x10^3/mm^3$)	0.102 ± 0.036	0.063 ± 0.023*	0.104 ± 0.041	0.051 ± 0.023**
	10	10	10	7

*P<0.05, **P<0.01 vs control group.
When significant (P<0.05) difference was found in one-way ANOVA, Dunnett's test was followed for comparisons between the vehicle control and treated groups.

Blood biochemistry

Sex: Male Stage: Week 4

Dose (mg/kg)	0 (control)	30	100	300
Number of Animals	10	10	10	10
	Mean ± SD	Mean ± SD	Mean ± SD	Mean ± SD
Item (unit)	n	n	n	n
AST (IU/L)	59.1 ± 4.3	67.2 ± 6.5	70.9 ± 13.3	80.6 ± 15.2**
	10	10	10	10
ALT (IU/L)	23.9 ± 3.6	24.4 ± 1.3	26.3 ± 3.9	39.8 ± 9.2**
	10	10	10	10
ALP (IU/L)	547.4 ± 111.3	545.8 ± 129.3	563.6 ± 102.3	830.6 ± 230.7**
	10	10	10	10
TBI (mg/dL)	0.02 ± 0.04	0.01 ± 0.03	0.04 ± 0.05	0.10 ± 0.05**
	10	10	10	10
CHO (mg/dL)	58.1 ± 8.4	64.3 ± 10.1	67.5 ± 12.8	85.5 ± 22.1**
	10	10	10	10
PL (mg/dL)	92.0 ± 10.1	100.2 ± 12.5	95.7 ± 13.4	110.6 ± 19.7*
	10	10	10	10
BUN (mg/dL)	15.11 ± 2.85	15.01 ± 2.17	15.82 ± 2.03	19.50 ± 2.45**
	10	10	10	10

Sex: Female Stage: Week 4

Dose (mg/kg)	0 (control)	30	100	300
Number of Animals	10	10	10	15
	Mean ± SD	Mean ± SD	Mean ± SD	Mean ± SD
Item (unit)	n	n	n	n
ALT (IU/L)	17.1 ± 2.0	18.3 ± 3.7	17.0 ± 1.8	26.0 ± 12.4*
	10	10	10	7
ALP (IU/L)	289.0 ± 35.8	273.0 ± 51.9	278.9 ± 64.4	421.1 ± 88.4**
	10	10	10	7
TBI (mg/dL)	0.01 ± 0.03	0.04 ± 0.05	0.07 ± 0.05	0.20 ± 0.10**
	10	10	10	7
CHO (mg/dL)	63.4 ± 10.8	63.7 ± 13.9	74.8 ± 10.0	109.6 ± 19.0**
	10	10	10	7
PL (mg/dL)	112.4 ± 15.6	108.7 ± 17.1	121.3 ± 13.1	158.9 ± 19.2**
	10	10	10	7
TG (mg/dL)	16.2 ± 8.0	16.5 ± 9.2	23.4 ± 10.7	52.6 ± 27.7**
	10	10	10	7

*P<0.05, **P<0.01 vs control group.
When significant (P<0.05) difference was found in one-way ANOVA, Dunnett's test was followed for comparisons between the vehicle control and treated groups.

Supplementary Table 27 Inhibitory effect of OPC-163493 on radioligand binding to various receptors, ion channels and transporters

Assay name	Inhibition (%)	
	OPC-163493	Positive substance
α1A-Adrenergic	0.00	100.00 (Prazosin)
α1B-Adrenergic	3.63	100.00 (Prazosin)
α2A-Adrenergic (Human)	0.00	100.00 (Rauwolscine)
α2B-Adrenergic (Human)	0.00	100.00 (Rauwolscine)
α2C-Adrenergic (Human)	0.00	100.00 (Rauwolscine)
β1-Adrenergic (Human)	8.84	99.70 ((±)-Propranolol)
β2-Adrenergic (Human)	0.00	99.58 ((±)-Propranolol)
Bradykinin B1 (Human)	22.68	100.00 (Lys-(des-Arg^9, Leu^8)-Bradykinin)
Bradykinin B2 (Human)	3.90	99.28 (HOE140)
Ca Channel (Type L, Benzothiazepine)	0.00	94.41 ((+)-*cis*-Diltiazem)
Ca Channel (Type L, Dihydropyridine)	4.23	98.75 (Nitrendipine)
Ca Channel (Type L, Phenylalkylamine)	0.00	95.22 ((±)-Methoxyverapamil)
Ca Channel (Type N)	24.23	100.00 (ω-Conotoxin GVIA)
Cannabinoid CB1 (Human)	1.33	94.58 ((*R*)-(+)-WIN55212-2)
Cannabinoid CB2 (Human)	14.15	100.00 ((*R*)-(+)-WIN55212-2)
CRF1 (Human)	0.00	99.95 (Urocortin human)
Dopamine D1 (Human)	0.00	100.00 (*R*(+)-SCH-23390)
Dopamine D2 short (Human)	0.00	100.00 ((+)-Butaclamol)
Dopamine D3 (Human)	27.27	100.00 ((±)-7-OH-DPAT)
Dopamine D4.2 (Human)	0.00	94.38 (Haloperidol)
Dopamine D5 (Human)	35.04	100.00 (*R*(+)-SCH-23390)
Dopamine transporter (Human)	1.32	100.00 (GBR12909)
Endothelin ETA (Human)	46.60	98.77 (Endothelin-1)
Endothelin ETB (Human)	1.84	100.00 (Endothelin-1)
GABA A (Agonist site)	0.00	94.65 (Muscimol)
GABA A (BZ central)	2.33	100.00 (Diazepam)
GABA A (Chloride channel)	6.20	100.00 (Picrotoxin)
GABA B	4.36	99.63 (GABA)
GABA transporter	7.24	100.00 (GABA)
Glucocorticoid (Human)	2.20	100.00 (Dexamethasone)

Supplementary Table 27 Continued

Assay name	Inhibition (%)	
	**OPC-163493**	**Positive substance**
Glutamate (AMPA)	0.00	99.97 ((S)-AMPA)
Glutamate (Kainate)	9.51	95.91 (Kainic acid)
Glutamate (NMDA agonist site)	4.93	98.95 (L-Glutamic acid)
Glutamate (NMDA glycine site)	22.46	95.18 (MDL105,519)
Histamine H1 (Human)	0.00	99.82 (Pyrilamine)
Histamine H2 (Human)	0.00	98.99 (Cimetidine)
Histamine H3 (Human)	0.00	98.95 ((R)(−)-α-Methylhistamine)
K Channel KATP	3.56	100.00 (Glibenclamide)
K Channel SkCa	14.73	100.00 (Apamin)
Leukotriene B4	6.22	96.38 (Leukotriene B_4)
Leukotriene D4	14.68	99.24 (Leukotriene D_4)
Melatonin MT1 (Human)	5.36	100.00 (Melatonin)
Muscarinic M1 (Human)	12.96	100.00 (Atropine)
Muscarinic M2 (Human)	16.10	100.00 (Atropine)
Muscarinic M3 (Human)	2.92	100.00 (Atropine)
Muscarinic M4 (Human)	4.36	100.00 (Atropine)
Muscarinic M5 (Human)	1.86	99.75 (Atropine)
Neurokinin NK1 (Human)	17.28	98.61 (L-703,606)
Neurokinin NK2 (Human)	8.32	100.00 (Neurokinin A)
Nicotinic (Human)	17.42	100.00 ((±)-Epibatidine)
Opiate δ (Human)	11.55	99.84 (Naltriben)
Opiate κ (Human)	9.14	100.00 (U-69593)
Opiate μ (Human)	15.33	100.00 (DAMGO)
Opiate ORL1 (Human)	10.49	98.21 (Nociceptin human)
Prostanoid EP2 (Human)	0.00	100.00 (Prostaglandin E2)
Serotonin 5HT1A (Human)	11.79	97.95 (Serotonin)
Serotonin 5HT2A (Human)	0.00	99.23 (Ketanserin)
Serotonin 5HT3 (Human)	0.00	100.00 (Tropisetron)
Serotonin transporter (Human)	0.00	99.43 (Imipramine)

Test substance concentration: 1×10^{-5} M, Positive substance concentration: 1×10^{-6} or 1×10^{-5} M. Data are expressed as the mean values of duplicate samples. The inhibition ratio was calculated from “100–binding ratio,” binding ratio: $[(B - N) / (B_0 - N)] \times 100$ (%), B: Bound radioactivity in the presence of test substance (individual value), B_0: Total bound radioactivity in the absence of test substance (mean value), N: Non-specific bound radioactivity (mean value).

Supplementary Discussions

Supplementary Discussion 1: Speculating further, SDH might be partly reversed in the direction of reducing fumarate to succinate, because it consequently provides oxidized Coenzyme Q to Complex I and promotes the circulation of the quinone pool[1]. As an anaplerotic response to the TCA cycle alteration, aspartate utilization for replenishment was in particular markedly activated. As a similar response, it was reported that aspartate utilization is essential for survival and growth of cancer cells when the TCA cycle was disrupted by the inhibition of 2-OG dehydrogenase [2].

Supplementary Discussion 2: Superoxide reacts with NO to form peroxynitrite ($ONOO^-$), which reduces NO bioavailability. $ONOO^-$ itself also oxidizes tetrahydrobiopterin (BH4), an essential cofactor of endothelial NO synthase (eNOS). This oxidization causes dysregulation of eNOS, known as eNOS uncoupling (different from mUncoupling). This in turn results in impaired NO release and leads to further superoxide production, setting up a vicious cycle and subsequent endothelial dysfunction[3].

Supplementary References

1. Chouchani, E.T., *et al.* Ischaemic accumulation of succinate controls reperfusion injury through mitochondrial ROS. *Nature* **515**, 431-435 (2014).
2. Allen, E.L., *et al.* Differential Aspartate Usage Identifies a Subset of Cancer Cells Particularly Dependent on OGDH. *Cell Rep* **17**, 876-890 (2016).
3. Kietadisorn, R., Juni, R.P. & Moens, A.L. Tackling endothelial dysfunction by modulating NOS uncoupling: new insights into its pathogenesis and therapeutic possibilities. *Am J Physiol Endocrinol Metab* **302**, E481-495 (2012).
