# Supplementary material for: Antidiabetic and cardiovascular beneficial effects of a liver-localized mitochondrial uncoupler
Source: Nat Commun. 2019 May 15;10:2172. doi: 10.1038/s41467-019-09911-6 (PMC6520346; doi:10.1038/s41467-019-09911-6)
Supplement: Supplementary file 3 — Reporting Summary [file 41467_2019_9911_MOESM3_ESM.pdf]

nature research

Corresponding author(s): Naohide Kanemoto
NCOMMS-17-34126C

Reporting Summary

Nature Research wishes to improve the reproducibility of the work that we publish. This form provides structure for consistency and transparency in reporting. For further information on Nature Research policies, see Authors & Referees and the Editorial Policy Checklist.

Statistical parameters

When statistical analyses are reported, confirm that the following items are present in the relevant location (e.g. figure legend, table legend, main text, or Methods section).

n/a	Confirmed	
☐	☒	The exact sample size (*n*) for each experimental group/condition, given as a discrete number and unit of measurement
☒	☐	An indication of whether measurements were taken from distinct samples or whether the same sample was measured repeatedly
☐	☒	The statistical test(s) used AND whether they are one- or two-sided<br>*Only common tests should be described solely by name; describe more complex techniques in the Methods section.*
☐	☒	A description of all covariates tested
☐	☒	A description of any assumptions or corrections, such as tests of normality and adjustment for multiple comparisons
☐	☒	A full description of the statistics including central tendency (e.g. means) or other basic estimates (e.g. regression coefficient) AND variation (e.g. standard deviation) or associated estimates of uncertainty (e.g. confidence intervals)
☐	☒	For null hypothesis testing, the test statistic (e.g. *F*, *t*, *r*) with confidence intervals, effect sizes, degrees of freedom and *P* value noted<br>*Give P values as exact values whenever suitable.*
☒	☐	For Bayesian analysis, information on the choice of priors and Markov chain Monte Carlo settings
☒	☐	For hierarchical and complex designs, identification of the appropriate level for tests and full reporting of outcomes
☒	☐	Estimates of effect sizes (e.g. Cohen's *d*, Pearson's *r*), indicating how they were calculated
☐	☒	Clearly defined error bars<br>*State explicitly what error bars represent (e.g. SD, SE, CI)*

Our web collection on statistics for biologists may be useful.

Software and code

Policy information about availability of computer code

Data collection	n/a
Data analysis	Statistic analysis: SAS software (R9.3, SAS Institute Japan)

For manuscripts utilizing custom algorithms or software that are central to the research but not yet described in published literature, software must be made available to editors/reviewers upon request. We strongly encourage code deposition in a community repository (e.g. GitHub). See the Nature Research guidelines for submitting code & software for further information.

Data

Policy information about availability of data

All manuscripts must include a data availability statement. This statement should provide the following information, where applicable:

- Accession codes, unique identifiers, or web links for publicly available datasets
- A list of figures that have associated raw data
- A description of any restrictions on data availability

Data availability
All data relevant to the manuscript are available from the corresponding author upon reasonable request.
The data that support the finding of this study are presented in this published article, its supplementary information, source data file and Public databases*.

The source data underlying Figs 1c, e-j, 2a-i, 3a-f, 4b, 8b-d, 9a, b, Supplementary Figs 1a, c, d, 2a, c, 3a-n, q-w, 4, 5a, b, 6a-e and Supplementary Tables 3-26 are provided as a Source Data file.
*Metabolomics data were deposited in the Metabolomics Workbench or the MetaboLights.
Accession code: ST001135 [http://dx.doi.org/10.21228/M8FH6C]
Accession code: ST001136 [http://dx.doi.org/10.21228/M8FH6C]
Accession code: MTBLS836 [https://www.ebi.ac.uk/metabolights/]
A reporting summary for this Article is available as a supplementary Information file

Field-specific reporting

Please select the best fit for your research. If you are not sure, read the appropriate sections before making your selection.

☒ Life sciences ☐ Behavioural & social sciences ☐ Ecological, evolutionary & environmental sciences

For a reference copy of the document with all sections, see nature.com/authors/policies/ReportingSummary-flat.pdf

Life sciences study design

All studies must disclose on these points even when the disclosure is negative.

Sample size	Sample size was estimated based on our preliminary experiments and previous experience. Especially in each animal study, animal number, which was likely to be the minimum required number, was carefully determined after discussion with animal care and use committee in Otsuka.
Data exclusions	In the 10-week dosing study with mixed chow in ob/ob mice, since an animal in control chow group showed 20% of body weight reduction with poor condition during the experiment, the animal was excluded and euthanized in accordance with Otsuka's guidelines.
Replication	All experiments were replicated twice or more except three long-term animal studies using old ZDF rats, OLETF rats, and ob/ob mice.
Randomization	Each group allocation was performed by the stratified randomization method on the basis of necessary baseline data.
Blinding	The investigators were not blinded to allocation during experiments.

Reporting for specific materials, systems and methods

Materials & experimental systems

n/a	Involved in the study	
☒	☐	Unique biological materials
☐	☒	Antibodies
☐	☒	Eukaryotic cell lines
☒	☐	Palaeontology
☐	☒	Animals and other organisms
☒	☐	Human research participants

Methods

n/a	Involved in the study	
☒	☐	ChIP-seq
☒	☐	Flow cytometry
☒	☐	MRI-based neuroimaging

Antibodies

Antibodies used	All antibodies used in western blotting were commercially available ones (Cell Signaling Technology).<br>Anti-AMPK-α (#2532), Anti-Phospho-AMPKα (Thr172) (#2531), Anti-Acetyl-CoA carboxylase (#3662), Anti-Phospho-Acetyl-CoA carboxylase (Ser79) (#3661), and Anti-rabbit IgG, HRP-linked (#7074).
Validation	All antibodies were validated by the suppliers (Cell Signaling Technology).

Eukaryotic cell lines

Policy information about cell lines

Cell line source(s)	Cell lines used were obtained either from Otsuka cell bank or directly purchased from DS Pharma.<br>Hep G2, DS Pharma #EC85011430<br>CHO-K1, DS Pharma #EC85051005

	Control CHO, Otsuka cell bank<br>NaCT-CHO, Otsuka cell bank
Authentication	No cell line authentication was performed by the authors.
Mycoplasma contamination	When we made cell stocks, cells were routinely tested for mycoplasma. After initiating frozen cell, the cell cultures were not maintained for long time (passages < 20).
Commonly misidentified lines<br>(See ICLAC register)	No commonly misidentified cell lines were used.

Animals and other organisms

Policy information about studies involving animals; ARRIVE guidelines recommended for reporting animal research

Laboratory animals	Only male animals were used in all animal experiments except toxicity study and QWBA study which used normal or pregnant female SD rats besides male rats.<br>Sprague-Dawley (SD) rats: Crl:CD(SD), Charles River Laboratories Japan, rearing period: age from 6 to 20 weeks.<br>ZDF rats: ZDF-Leprfa/CrlCrlj, Charles River Laboratories Japan, rearing period: age from 5 to 33 weeks.<br>ZDF(M) rats: ZDF(M)-Leprfa/CrlCrlj, Charles River Laboratories Japan, rearing period: age from 4 to 11 weeks.<br>ob/ob mice: B6.V-Lep ob /J, Charles River Laboratories Japan, rearing period: age from 5 to 18 weeks.<br>Akita mice: AKITA/Slc, Japan SLC, rearing period: age from 6 to 12 weeks.<br>SHRSPs: SHRSP/Izm, Japan SLC, rearing period: age from 5 to 18 weeks.<br>OLETF rats: Otsuka Long-Evans Tokushima Fatty, Hoshino Laboratory Animals, rearing period: age from 5 to 47 weeks.<br>LETO rats: Long-Evans Tokushima Otsuka, Hoshino Laboratory Animals, rearing period: age from 5 to 47 weeks.
Wild animals	n/a
Field-collected samples	n/a
